# Body mass index and incident cardiometabolic conditions in relation to obesity‐related cancer risk: A population‐based cohort study in Catalonia, Spain

**DOI:** 10.1002/cam4.6603

**Published:** 2023-09-28

**Authors:** Martina Recalde, Andrea Pistillo, Vivian Viallon, Emma Fontvieille, Talita Duarte‐Salles, Heinz Freisling

**Affiliations:** ^1^ Fundació Institut Universitari per a la recerca a l'Atenció Primària de Salut Jordi Gol i Gurina (IDIAPJGol) Barcelona Spain; ^2^ Universitat Autònoma de Barcelona Bellaterra Spain; ^3^ International Agency for Research on Cancer (IARC‐WHO) Lyon Cedex France

**Keywords:** adiposity, cancer, cardiovascular disease, electronic health record, hypertension, type 2 diabetes, multimorbidity

## Abstract

**Background:**

We investigated the association between body mass index (BMI) and obesity‐related cancer risk among individuals with/without incident hypertension (HTN), type 2 diabetes mellitus (T2DM), and cardiovascular disease (CVD) and the joint associations of overweight/obesity (BMI ≥25 kg/m^2^) and each cardiometabolic condition with obesity‐related cancer risk

**Methods:**

We conducted a population‐based cohort (*n* = 1,774,904 individuals aged ≥40 years and free of cancer and cardiometabolic conditions at baseline) study between 2010 and 2018 with electronic health records from Spain. Our main outcome measures were hazard ratios (HRs) for incident obesity‐related cancers and relative excess risk due to interaction (RERI).

**Results:**

A total of 38,082 individuals developed obesity‐related cancers after a median of 8 years of follow‐up. The positive association between BMI and obesity‐related cancer risk was similar among individuals free of cardiometabolic conditions (hazard ratio, HR per 5 kg/m^2^: 1.08, 95% confidence interval, CI: 1.06–1.10) and with incident HTN (1.05, 1.01–1.08). The association among those with incident T2DM was null (0.98, 0.93–1.03). There was a positive additive interaction between overweight/obesity and CVD (relative excess risk due to interaction [RERI]: 0.19 [0.09, 0.30]), meaning that the combined association was 0.19 more than the sum of the individual associations. In contrast, a RERI of −0.24 (−0.28, −0.20) was observed for the combined association between overweight/obesity and T2DM.

**Conclusions:**

Public health strategies to reduce overweight can help prevent cancer cases among the general population and individuals with incident HTN/CVD. Further, weight‐loss interventions seem to lead to a greater cancer risk reduction among individuals with CVD.

## Graphical abstract

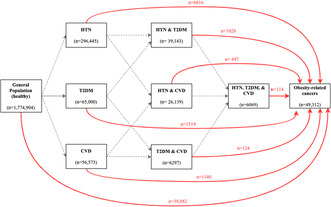

Disease trajectories as indicated by red arrows were investigated for the associations between BMI and obesity‐related cancer risk. A BMI increment of 5 kg/m2 in multivariable‐adjusted models was associated with an 8% (HR: 1.08, 95% CI: 1.06‐1.10) higher relative risk of obesity‐related cancers among “healthy” individuals and a 5% higher relative risk among those with HTN (HR: 1.05, 95%CI: 1.01‐1.08). The HRs for the remaining transitions were as follows: CVD (1.08, 0.97‐1.21), HTN, T2DM, & CVD (1.05, 0.82‐1.33), HTN & CVD (1.03, 0.92‐1.15), T2DM & CVD (1.02, 0.84‐1.24), HTN & T2DM (1.00, 0.93‐1.07) and T2DM (0.98, 0.93‐1.03) (in descending order of effect size).

## 1 INTRODUCTION

The prevalence of overweight (body mass index, BMI ≥25 & <30 kg/m^2^) and obesity (BMI ≥30 kg/m^2^) has rapidly risen over the past decades, reaching more than 1.9 billion and 650 million adults in 2016, respectively.^1^ A high BMI (a proxy of general adiposity) has been convincingly associated with at least 13 cancer types (labeled as obesity‐related cancers).^2^ It has also been associated with a higher risk of cardiometabolic conditions such as hypertension (HTN), type 2 diabetes mellitus (T2DM), and cardiovascular diseases (CVD).^3^ The prevalence of these conditions has highly increased over the past decades.^4^, ^5^, ^6^ Moreover, HTN and T2DM have been proposed as risk factors for certain cancers.^7^ CVD and cancer have been shown to share common molecular pathways,^7^, ^8^ and emerging evidence also suggests that CVD might be an independent risk factor for cancer.^9^

The co‐presence of cardiometabolic conditions and adiposity may interact synergistically and potentially exacerbate cancer risk associated with obesity. However, the extent to which these cardiometabolic conditions modify the BMI‐cancer association has largely not been investigated in prior studies.^10^, ^11^, ^12^ In addition, prior studies have not investigated the combination of component risk factors (i.e., adiposity and cardiometabolic conditions) in relation to cancer risk. It is important to address whether BMI‐cancer associations differ among population groups affected by cardiometabolic conditions, especially given the rise in their prevalence. From a public health and clinical perspective, such knowledge could help target population groups that could be prioritized in lifestyle interventions or cancer screening programs. How co‐present, or sequential diseases and related risk factors promote negative effects of disease interaction has been referred to as the syndemics model of health.^13^

In this work, we investigated whether incident HTN, T2DM, or CVD modify the association between BMI and the risk of developing obesity‐related cancers (primary aim), using electronic health record (EHR) data from Catalonia, Spain. Our secondary aim was to study the joint associations of overweight/obesity and incident cardiometabolic conditions with obesity‐related cancer risk.

## 2 METHODS

### 2.1 Study design, setting, and data sources

We conducted a population‐based cohort study from January 1, 2010 to December 31, 2018 using prospectively collected primary care records from the Information System for Research in Primary Care (SIDIAP; www.sidiap.org) in Catalonia, Spain. The SIDIAP is a pseudo‐anonymized database of EHRs containing data from 5.8 million people living in Catalonia since 2006. This database covers >75% of the population of Catalonia and is representative of the overall population in terms of age, sex, and geographic distribution.^14^ The SIDIAP contains data on anthropometric measurements, disease diagnoses (International Classification for Diseases, 10th revision [ICD‐10]), and demographic and lifestyle information, among others. Further, SIDIAP can be linked to the Minimum Basic Dataset (CMBD in Spanish), a population‐based registry of hospital discharge information including diagnoses and procedures.^15^

### 2.2 Participants

**FIGURE 1:**
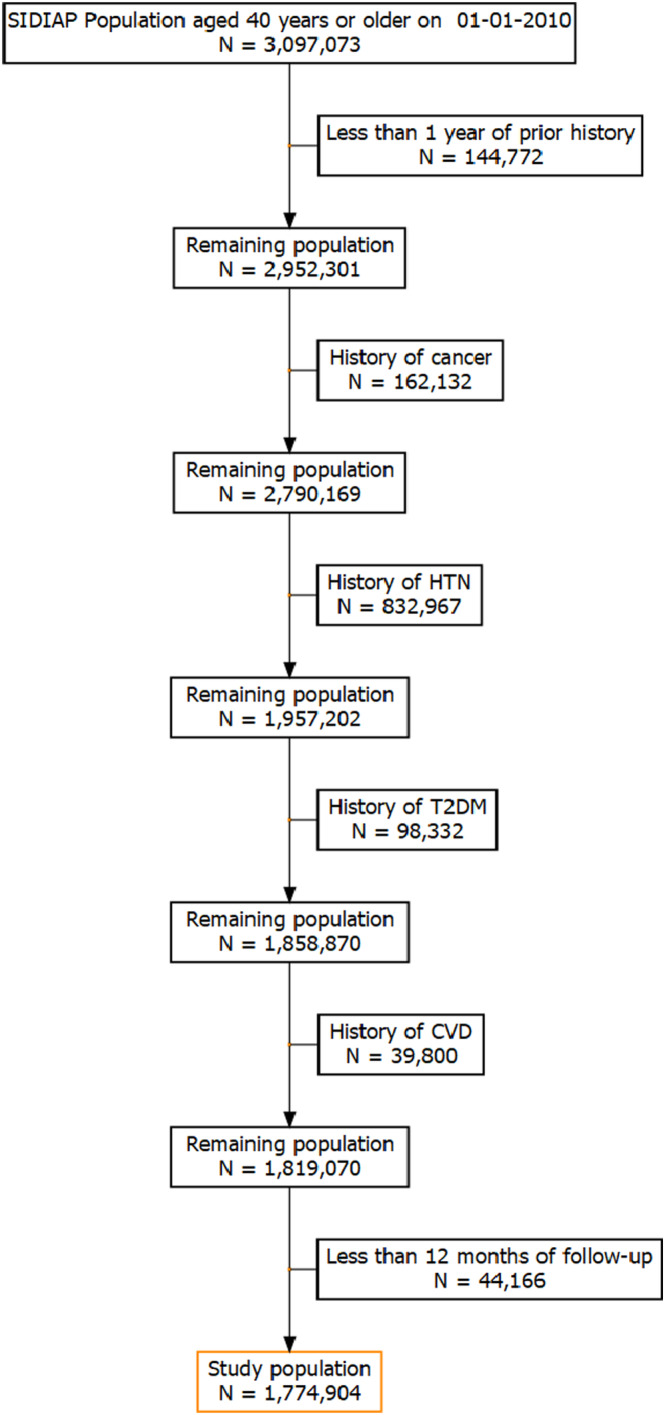
Flowchart with the inclusion and exclusion criteria of the study participants. History of cancer considers any type of cancer (C00‐C97) except other and unspecified malignant neoplasm of skin (C44). Causes of end‐of‐follow‐up include transferral out of SIDIAP, cancer diagnosis, death, or end‐of‐study period. Individuals with less than 12 months of follow‐up were excluded because the follow‐up of the participants started 1 year after study entry to avoid potential reverse causality (e.g., BMI affected by undiagnosed cancer). The proportion of excluded individuals due to prevalent cancer or the cardiometabolic conditions should not be interpreted as the proportion of individuals with that specific prevalent disease/condition in SIDIAP given the sequence of excluding participants with prevalent conditions and the overlap between individuals with more than one disease/condition. CVD, Cardiovascular disease; HTN, Hypertension; SIDIAP, Information System for Research in Primary Care; T2DM, Type 2 diabetes mellitus.

We included all individuals aged ≥40 years registered in SIDIAP on January 1, 2010 (index date). We excluded participants who had been registered in the database for less than one year, who had been diagnosed with any cancer type (except other and unspecified malignant neoplasm of skin), HTN, T2DM, and/or CVD prior to index date, and those with less than 12 months follow‐up (Figure 1). The follow‐up period extended between 1 year after index date (to minimize the possibility of reverse causality [i.e., BMI affected by undiagnosed cancer]) and exit from the database, death, cancer diagnosis (any except other and unspecified malignant neoplasm of skin), or the end of study period (December 31, 2018), whichever occurred first.

### 2.3 Outcome assessment

The outcome was a binary indicator of incident diagnoses of a first primary obesity‐related cancer which we identified with ICD‐10 and ICD‐9 codes in the SIDIAP and CMBD hospital discharge databases, respectively (Table S1). Obesity‐related cancers comprised cancers of the colorectum; liver; gallbladder and biliary tract; pancreas; post‐menopausal breast; corpus uteri; ovary; kidney; brain and central nervous system; thyroid; and multiple myeloma.^2^

We stratified breast cancers at the time of diagnosis into pre‐ and postmenopausal based on information registered in medical records between the ages of ≥45 and ≤ 55 years. In case no information regarding the menopausal status was available, we considered a woman to be postmenopausal if she was aged ≥50 years at the time of the breast cancer diagnosis.

We omitted esophagus and stomach cancers from the list of obesity‐related cancers given that with the available data we could not differentiate esophageal adenocarcinoma (obesity‐related) from squamous cell carcinoma or gastric cardia (obesity‐related) from non‐cardia cancers. The incidence of the non‐obesity‐related subtypes/subsites of these cancers is higher in Spain.^16^

In a previous study comparing cancer diagnoses registered in the SIDIAP including the CMBD to those of provincial population‐based registries of Catalonia, the sensitivity for obesity‐related cancers ranged between 74.6 (70.2–79.0) for thyroid cancer and 90.4 (89.4–91.4) for breast postmenopausal (only gallbladder & biliary tract fell outside this range 50.0 [42.6–57.4]).^17^

### 2.4 Exposures assessment

BMI (continuous in kg/m^2^) was calculated using weight (kg) and height (cm) assessed in a standardized manner by general practitioners or nurses.^18^ We implemented a multilevel time raster multiple imputation approach to have complete information on BMI for all study participants and to update BMI values every time a participant was diagnosed with a cardiometabolic condition or a combination of these conditions.^19^ We assumed that BMI assessments were missing at random (MAR) given that despite observing different characteristics between individuals with and without a BMI assessment, the distribution of BMI in SIDIAP was similar to population‐based surveys of the Spanish population.^12^

**FIGURE 2:**
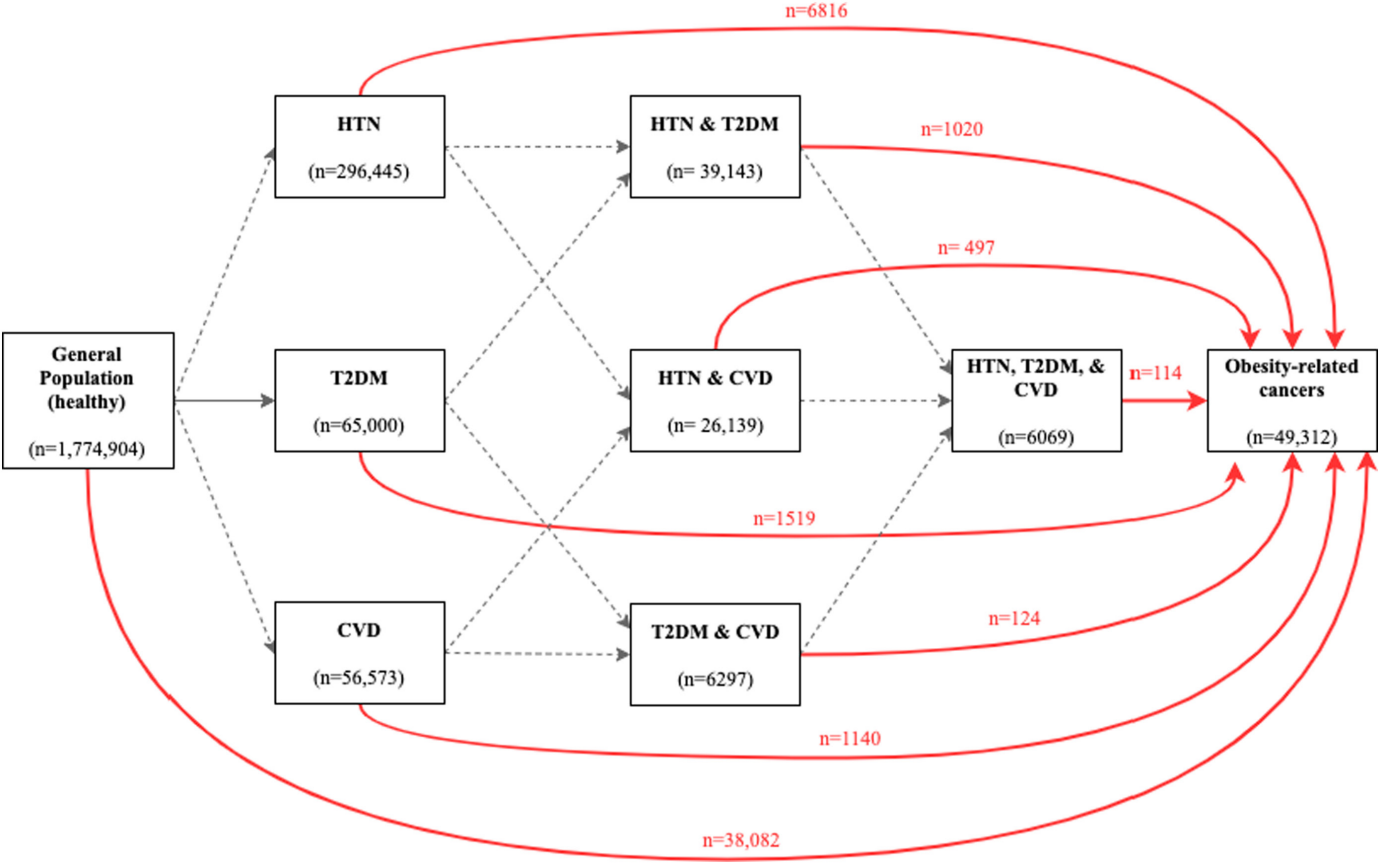
Framework for the definition of the time‐varying variable “cardiometabolic conditions”. Red arrows represent the disease‐trajectories that were the main interest of this study, while gray dashed lines represent the ones that were not investigated given the research question of this study. CVD, cardiovascular disease; HTN, hypertension; T2DM, type 2 diabetes mellitus.

For our secondary objective, the exposure was a composite variable of 16 categories combining binary BMI (< or ≥ 25 kg/m^2^) and cardiometabolic conditions, coded as a time‐varying variable with eight categories (“*healthy”*; *HTN*; *T2DM*; *CVD*; *HTN* & *T2DM*; *HTN* & *CVD*; *T2DM* & *CVD*; *HTN*, *T2DM*, & *CVD*). All study participants were in the “*healthy”* category at index date, and during follow‐up, they could change states as shown in Figure 2. We decided a priori to focus on individuals with incident cardiometabolic conditions and exclude those with prevalent conditions to minimize potential reverse causation of BMI (assessed at index date and during follow‐up) related to cardiometabolic conditions. HTN and T2DM were identified using diagnostic codes recorded in the SIDIAP database (Table S1). CVD was defined as any diagnosis of coronary or cerebrovascular disease which we identified using data from the CMBD hospital discharge and SIDIAP (Table S1).^20^ Further details on the definition of HTN, T2DM, and CVD and the respective ICD‐9 and ICD‐10 codes are given in Table S1.

### 2.5 Covariates of interest

The covariates were sex (*female*, *male*), age (in years and 5‐year categories) at index date (and updated at a diagnosis of a cardiometabolic condition), geographic region of nationality (*Spanish*, *Global North*, or *Global South*),^21^ and socioeconomic status (assessed using the *Mortalidad en áreas pequeñas Españolas y Desigualdades Socioeconómicas y Ambientales* (MEDEA) deprivation index (calculated at the census tract level and categorized into quintiles by the SIDIAP for anonymization purposes with urban areas as a separate category).^22^ We also extracted information on smoking status (*never*, *former*, or *current smoker*) and alcohol intake (*no*, *low*, or *high risk*) (the closest record to the index date within 5 years before or at the index date was selected).

### 2.6 Statistical analyses

We applied multilevel time raster multiple imputation to BMI at several time points (2006, 2010, 2013, 2016, 2018).^19^ We used a linear mixed‐effects model with five imputations to obtain imputed trajectories of BMI for the study participants (Appendix S1).^19^ BMI at baseline was defined as the corresponding value to the “2010” time point. For participants diagnosed with one or more cardiometabolic conditions, we updated their BMI measurement using the closest prior time point to the date of diagnosis. This approach was predefined in our statistical analysis plan and was intended to provide a more accurate association of current BMI and obesity‐related cancer risk.

To investigate if incident HTN, T2DM, or CVD modify the association between BMI and obesity‐related cancer risk, we fitted Cox proportional hazards models with age as the time metric including BMI, cardiometabolic conditions as a time‐varying variable, and an interaction of those with BMI. We fitted two models, a model adjusted by sex and stratified by age at index date (5‐year categories) (minimally adjusted) and one further adjusted by nationality, MEDEA deprivation index, smoking status, and alcohol intake (fully adjusted model). We used a directed acyclic graph (DAG) to guide our decisions on the control for confounding (Figure S1).^23^ Missing data at baseline for the MEDEA deprivation index, smoking status, and alcohol intake were imputed (using predictive mean matching, an imputation technique that estimates the likely values of missing data by matching to the observed values/data, with five imputations drawn) (Appendix S1). We accounted for potential non‐linearity in the BMI‐obesity‐related cancer association by fitting models with BMI as a linear term, with a polynomial of degree 2, and with restricted cubic splines (3, 4, or 5 knots as recommended by Harrell).^24^ We estimated hazard ratios (HRs) and their 95% confidence intervals (CIs) per 5 kg/m^2^ increment of BMI. We evaluated multiplicative interaction between BMI and cardiometabolic conditions by comparing the log‐likelihood of models with and without the interaction term. We checked the proportional hazards assumptions by visual inspection of survival curves. We conducted two supplementary analyses to contextualize our findings: stratification of the results by sex and age groups (aged <65 or ≥65 years) to assess potential effect modification and re‐running the main model analyzing site‐specific cancers (with ≥100 cancer cases) as outcomes. As sensitivity analyses, we re‐run the main model (i) without updating the BMI and age of participants, and without imputed BMI by (ii) including only individuals with a real BMI assessment at baseline (iii) or also during follow‐up. We (iv) added as an adjustment variable the number of visits to primary care centers (year before study entry or upon diagnosis of cardiometabolic condition[s]) to account for potentially different health attitudes of the participants. Results of these sensitivity analyses (i) to (iv) are shown in Figure S5. To address potential collider bias and residual confounding by smoking, we also re‐run the main model (v) among never smokers and (vi) with a negative control outcome (i.e., non‐obesity‐related cancers). Finally, to assess potential outcome misclassification we re‐run the analysis (vii) using a more restrictive definition of the outcome (for corpus uteri we considered only C54 and C54.1 as codes of interest, and we excluded brain and CNS cancer from the obesity‐related cancer definition, as only meningioma is considered an obesity‐related cancer in this broad group).^2^

For our secondary aim, we assessed the relative excess risk due to interaction (RERI) of obesity‐related cancers between overweight/obesity (BMI≥25 kg/m^2^) and incident cardiometabolic conditions (joint effects analysis).^25^ We fitted a Cox proportional hazards model with age as the time metric including the composite variable adjusted by sex, nationality, MEDEA deprivation index, smoking status, alcohol intake, and stratified by age.^26^ The RERI was calculated as ${\text{RERI}}_{\mathrm{RR}}={\mathrm{RR}}_{11}-{\mathrm{RR}}_{10}-{\mathrm{RR}}_{01}+1$, where _11_ denotes being exposed to both factors (e.g., overweight/obesity and *HTN*), _10_ to one factor (e.g., overweight/obesity), and _01_ to the other one (e.g., *HTN*). A RERI of 0 was considered a lack of additive interaction and 95% CIs were calculated as proposed by Hosmer and Lemeshow.^27^ We also estimated incidence rates per 1000 person‐years. As an analogous measure to the RERI, we calculated risk differences between observed and expected joint associations of being exposed to both overweight/obesity and the condition(s) of interest. This measure was calculated by subtracting the observed (risk of obesity‐related cancer among people with a BMI ≥25 kg/m^2^ and the condition of interest) from the expected joint association (risk of obesity‐related cancer among people with BMI <25 kg/m^2^ and the condition + people with BMI ≥25 kg/m^2^ and “healthy”—people with BMI <25 kg/m^2^ and “healthy”).

To provide a better understanding of this joint association, we also performed a model for the association between the cardiometabolic conditions (8‐category variable) and obesity‐related cancers separately (supplementary analysis). For this analysis, we also explored potential residual confounding by smoking and collider bias (among never smokers and non‐obesity‐related cancers as the outcome).

We used R version 4.0.1 for all the analyses. We obtained approval from the Clinical Research Ethics Committee of the IDIAPJGol (project code: 20/237‐P) to perform this study.

## 3 RESULTS

There were 3,097,073 adults aged ≥40 years at index date eligible to enter the study. We excluded 144,772 individuals due to having less than 1 year of prior clinical history; 1,133,231 due to prevalent cancer, HTN, T2DM, or CVD; and 44,166 due to less than 1 year of follow‐up (Figure 1). The characteristics of the individuals excluded at each step of the definition of the population are reported in Table S2. Overall, the initial population (*n* = 3,097,073) was older and presented with more comorbidities and complete information on the covariates of interest than the study population (*n* = 1,774,904).

Of the 1,774,904 study participants, 681,386 (39%) had a BMI assessment at baseline, 589,319 (33%) had at least one BMI assessment during follow‐up and 504,199 (28%) did not have any BMI assessment (Table S3). Age was similarly distributed in the three groups (median age was 53, 51, and 49 years, respectively) and so was BMI among those with an assessment at baseline and only during follow‐up (median of 27 kg/m^2^ for both). However, those without any BMI measurement had a higher representation of males, non‐Spanish individuals, individuals living in the least deprived areas, had fewer comorbidities, and were more frequently transferred out of SIDIAP than those with a BMI assessment at baseline.

Across all study participants, the median BMI at baseline was 27 (interquartile range [IQR]: 24–30) kg/m^2^ (after multiple imputations), the median age was 51 (44–60) years and 53% were females (Table 1). Compared to those living with obesity, those with normal or underweight, were more frequently females, living in the least deprived areas, and were current smokers.

**TABLE 1:** Baseline characteristics of the study participants by body mass index categories, after multiple imputations.

	Overall *N* (%)	By WHO categories of BMI^a^ *N* (%)		
		Normal or underweight	Overweight	Obesity
	1,774,904 (100.0)	606,249 (34.0)	722,839 (41.0)	445,816 (25.0)
Follow‐up time in years, median (IQR)	8.0 (8.0, 8.0)	8.0 (8.0, 8.0)	8.0 (8.0, 8.0)	8.0 (8.0, 8.0)
*N* of visits to primary care centers, median (IQR)	3.0 (0.0, 7.0)	2.0 (0.0, 6.0)	3.0 (0.0, 7.0)	3.0 (0.0, 8.0)
BMI in kg/m^2^, median (IQR)^b^	27.0 (23.9, 30.0)	23.0 (20.9, 23.9)	27.0 (26.2, 28.6)	32.0 (31.1, 34.6)
Age in years, median (IQR)	51.0 (44.0, 60.0)	50.0 (44.0, 59.0)	51.0 (45.0, 61.0)	51.0 (45.0, 61.0)
Female sex, *n* (%)	931,239 (52.5)	354,019 (58.4)	351,476 (48.6)	225,744 (50.6)
Nationality				
Spanish	1,632,639 (92.0)	561,797 (92.7)	665,784 (92.1)	405,057 (90.9)
Global North	48,735 (2.7)	17,109 (2.8)	18,982 (2.6)	12,643 (2.8)
Global South	93,530 (5.3)	27,342 (4.5)	38,072 (5.3)	28,116 (6.3)
MEDEA deprivation index, *n* (%)^b^				
Quintile 1 (least deprived)	334,723 (18.9)	131,872 (21.8)	133,284 (18.4)	69,567 (15.6)
Quintile 2	294,506 (16.6)	102,920 (17.0)	120,508 (16.7)	71,078 (15.9)
Quintile 3	278,367 (15.7)	91,423 (15.1)	114,662 (15.9)	72,281 (16.2)
Quintile 4	263,856 (14.9)	82,432 (13.6)	108,559 (15.0)	72,865 (16.3)
Quintile 5 (most deprived)	236,249 (13.3)	71,723 (11.8)	95,716 (13.2)	68,811 (15.4)
Rural	367,203 (20.7)	125,880 (20.8)	150,109 (20.8)	91,215 (20.5)
Smoking status, *n* (%)^b^				
Never smoker	1,090,923 (61.5)	362,602 (59.8)	448,072 (62.0)	280,249 (62.9)
Former smoker	205,295 (11.6)	67,046 (11.1)	84,940 (11.8)	53,308 (12.0)
Current smoker	478,686 (27.0)	176,601 (29.1)	189,827 (26.3)	112,259 (25.2)
Alcohol intake, *n* (%)^b^				
No risk	1,099,308 (61.9)	383,154 (63.2)	438,409 (60.7)	277,745 (62.3)
Low risk	602,673 (34.0)	199,364 (32.9)	254,167 (35.2)	149,142 (33.5)
High risk	72,923 (4.1)	23,732 (3.9)	30,262 (4.2)	18,929 (4.2)
Charlson comorbidity index, *n* (%)				
0	1,522,931 (85.8)	525,679 (86.7)	620,395 (85.8)	376,857 (84.5)
1	210,730 (11.9)	67,111 (11.1)	85,846 (11.9)	57,773 (13.0)
2	31,995 (1.8)	10,449 (1.7)	12,929 (1.8)	8617 (1.9)
≥3	9248 (0.5)	3010 (0.5)	3669 (0.5)	2570 (0.6)
Cause of exit from the study, *n* (%)				
End of study	1,373,650 (77.4)	467,137 (77.1)	562,394 (77.8)	344,119 (77.2)
Transferred out of the SIDIAP	219,024 (12.3)	77,669 (12.8)	868,880 (12.0)	54,474 (12.2)
Death	78,456 (4.4)	28,055 (4.6)	30,574 (4.2)	19,827 (4.4)
Obesity‐related cancers	49,312 (2.8)	15,318 (2.5)	20,016 (2.8)	13,978 (3.1)
Non‐obesity‐related cancers	54,462 (3.1)	18,069 (3.0)	22,974 (3.2)	13,419 (3.0)

Abbreviations: BMI, Body Mass Index; IQR, Interquartile range; MEDEA, “Mortalidad en áreas pequeñas Españolas y Desigualdades Socioeconómicas y Ambientales”; SIDIAP, Information System for Research in Primary Care; WHO, World Health Organization.

^a^ This categorization was done in the five datasets with the multiple imputations. For visualization purposes and in order for the categorical variables to add up to 1,774,904 we divided the *n* for the categorical variables by 5.

^b^ The statistics of BMI, the MEDEA deprivation index, smoking status, and alcohol intake were calculated using the multiple imputation approach, with five data sets created. For visualization purposes, we divided the *n* for the categorical variables by 5. BMI categories: underweight or normal weight [BMI <25 kg/m^2^], overweight [BMI ≥25 and < 30 kg/m^2^], and obesity [BMI ≥30 kg/m^2^]). Non‐obesity‐related cancers do not include non‐melanoma skin cancer.

After a median follow‐up of 8 years of the 1,774,904 (“*healthy”*) study participants, 38,082 (2.1%) were diagnosed with obesity‐related cancers (Figure 2 and Table S4). The proportion of obesity‐related cancers diagnosed among participants with one or more cardiometabolic condition was similar (Table S4).

### 3.1 Association of BMI with cancer risk by cardiometabolic conditions

**FIGURE 3:**
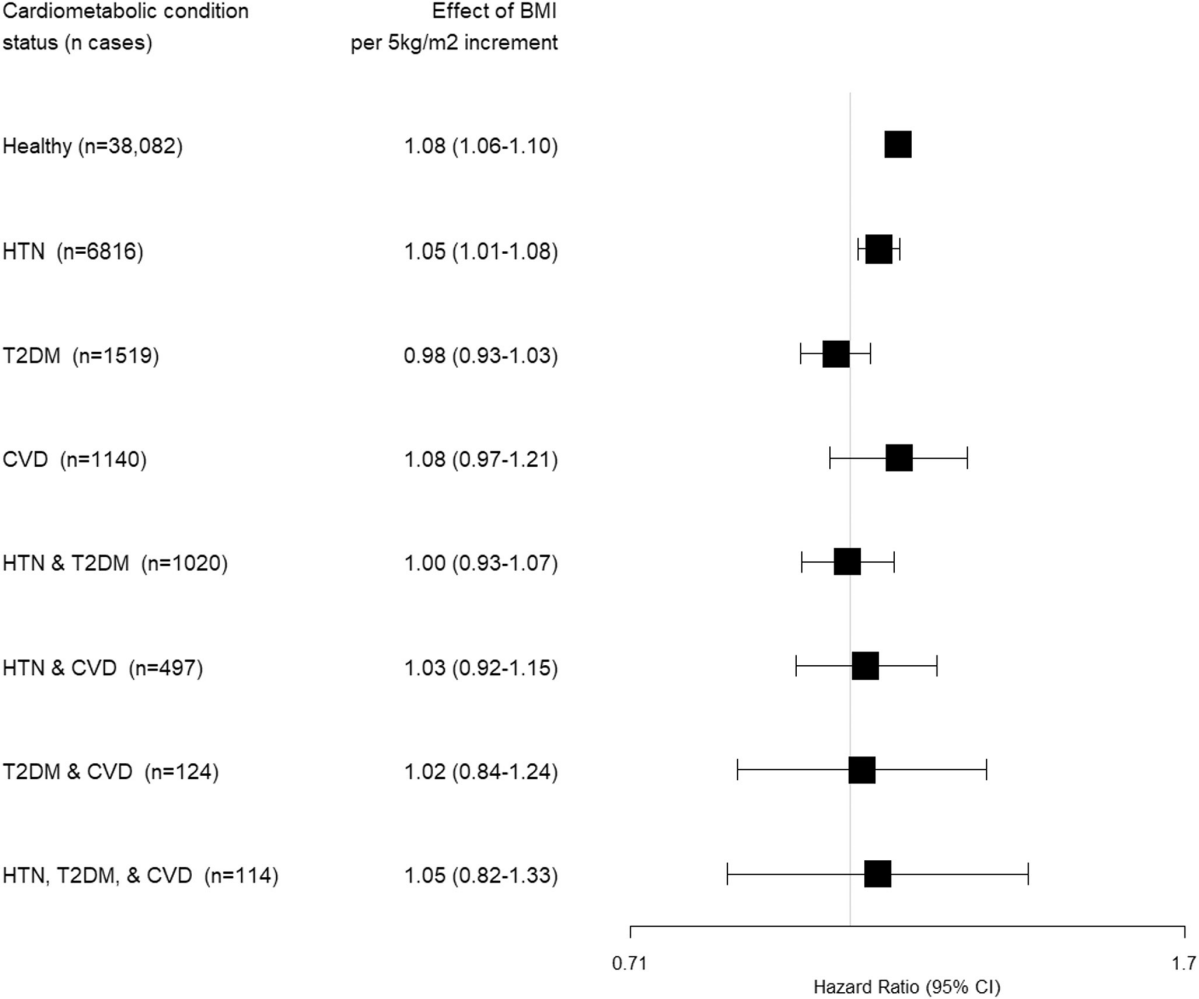
Hazard ratios (HR) with 95% confidence intervals (CI) for associations between body mass index (BMI) and the risk of obesity‐related cancers by cardiometabolic conditions. *N* cases are obesity‐related cancer cases. The model included BMI as a continuous variable with an interaction term with the time‐varying “cardiometabolic conditions” variable and was adjusted by sex, the geographic region of nationality, the MEDEA deprivation index, smoking status, alcohol intake, and stratified by age (5‐year categories). We evaluated the interaction between BMI and the variable of cardiometabolic conditions by comparing the difference in log‐likelihood of models with and without the interaction term (*p* = 0.007). The *p*‐values for the interaction between BMI and each cardiometabolic condition (as extracted directly from the model output) were: 0.067 (HTN), 0.001 (T2DM), 0.980 (CVD), 0.034 (HTN & T2DM), 0.373 (HTN & CVD), 0.577 (T2DM & CVD), 0.790 (HTN, T2DM, & CVD). BMI, body mass index; CI, confidence interval; CVD, Cardiovascular disease; HTN, Hypertension; T2DM, Type 2 diabetes mellitus.

There was multiplicative interaction between BMI and cardiometabolic conditions (*p*‐value from log‐likelihood ratio test = 0.007) in the association with obesity‐related cancers (Figure 3). We did not find evidence of non‐linearity between BMI and cancer risk. A BMI increment of 5 kg/m^2^ in the main models was associated with an 8% (HR: 1.08, 95% CI: 1.06–1.10) higher relative risk of obesity‐related cancers among “*healthy”* individuals and 5% higher among those with *HTN* (HR: 1.05, 95% CI: 1.01–1.08) (Figure 3). Even though the CIs of the HRs for *CVD* (1.08, 0.97–1.21), *HTN*, *T2DM*, & *CVD* (1.05, 0.82–1.33), *HTN* & *CVD* (1.03, 0.92–1.15), and *T2DM* & *CVD* (1.02, 0.84–1.24) (in descending order of estimates) were positive, they overlapped with 1. We did not find evidence of interaction between these and BMI (p‐values for interaction >0.05) suggesting no differences in relative risks as compared to “healthy” individuals. In contrast, the HRs for associations for those with *T2DM* (0.98, 0.93–1.03) and *HTN* & *T2DM* (1.00, 0.93–1.07) were attenuated (*p*‐values for interaction were 0.001 and 0.034, respectively). The risk estimates in the minimally adjusted models were similar to those of the fully adjusted models (Figure S2). The results of the supplementary and sensitivity analyses are described in Appendix S2 and reported in Figures S3A,B–S5, and Tables S5–S7. The results of these additional analyses were in line with our expectations and confirmed our main analysis. For example, the point estimates among never smokers were reassuringly similar to our main analysis (Table S5).

### 3.2 Joint associations of overweight/obesity and cardiometabolic conditions with cancer risk

Compared to participants with a BMI < 25 kg/m^2^ and without a cardiometabolic condition (“*healthy*”), participants with overweight/obesity (BMI≥25 kg/m^2^) and with or without a cardiometabolic condition had a consistently higher relative risk of obesity‐related cancer (Table 2A). These results were consistent (but stronger in magnitude) with those of the supplementary analysis investigating the association between cardiometabolic conditions and obesity‐related cancers (adjusting for continuous BMI) (Figure S6). There was evidence of additive interaction (RERI ≠0) for five out of seven joint associations of overweight/obesity and cardiometabolic conditions with obesity‐related cancer risk. For example, the RERI for the joint association of overweight/obesity and CVD was 0.19 [0.09, 0.30]. In contrast, the RERI for the joint association of overweight/obesity and T2DM was −0.22 [−0.29, −0.16] (Table 2A). The results of the sensitivity analyses among never smokers and with non‐obesity‐related cancer as a negative control outcome confirmed our main analyses and are described in Appendix S2 and reported in Tables S8 and S9.

**TABLE 2:** Joint association of overweight/obesity (BMI≥25 kg/m^2^) and cardiometabolic conditions with obesity‐related cancer risk

A. Hazard ratios (HR) and relative excess risk due to interaction (RERI)														
	HTN		T2DM		CVD		HTN & T2DM		HTN & CVD		T2DM & CVD		HTN, T2DM, & CVD	
	*n* at risk (*n* cases)	HR (95% CI)	*n* at risk (n cases)	HR (95% CI)	*n* at risk (*n* cases)	HR (95% CI)	*n* at risk (*n* cases)	HR (95% CI)	*n* at risk (*n* cases)	HR (95% CI)	*n* at risk (*n* cases)	HR (95% CI)	*n* at risk (*n* cases)	HR (95% CI)
BMI < 25 kg/m^2^, *“healthy”*	606,249 (12,861)	1 (ref)	606,249 (12,861)	1 (ref)	606,249 (12,861)	1 (ref)	606,249 (12,861)	1 (ref)	606,249 (12,861)	1 (ref)	606,249 (12,861)	1 (ref)	606,249 (12,861)	1 (ref)
BMI ≥ 25 kg/m^2^, *“healthy”*	1,168,655 (25,221)	1.11 (1.06–1.16)	1,168,655 (25,221)	1.11 (1.06–1.16)	1,168,655 (25,221)	1.11 (1.06–1.16)	1,168,655 (25,221)	1.11 (1.06–1.16)	1,168,655 (25,221)	1.11 (1.06–1.16)	1,168,655 (25,221)	1.11 (1.06–1.16)	1,168,655 (25,221)	1.11 (1.06–1.16)
BMI < 25 kg/m^2^, with condition	67,488 (1544)	1.40 (1.29–1.53)	10,345 (262)	2.18 (1.91–2.50)	17,056 (310)	1.76 (1.44–2.16)	4638 (132)	2.25 (1.82–2.77)	6590 (116)	1.63 (1.35–1.96)	1206 (20)	2.09 (1.18–3.71)	1023 (17)	1.76 (0.87–3.54)
BMI ≥ 25 kg/m^2^, with condition (joint effect)	228,957 (5272)	1.49 (1.44–1.55)	54,655 (1257)	2.07 (1.93–2.22)	39,517 (830)	2.07 (1.84–2.32)	34,505 (888)	2.12 (1.97–2.27)	19,549 (381)	1.78 (1.60–1.98)	5091 (104)	2.57 (2.08–3.17)	5046 (97)	2.10 (1.70–2.59)
RERI		−0.02 (−0.06, 0.02)		−0.22 (−0.29, −0.16)		0.19 (0.09, 0.30)		−0.24 (−0.28, −0.20)		0.04 (−0.07, 0.15)		0.36 (0.32, 0.40)		0.23 (0.02, 0.44)

B. Incidence rates per 1000 person‐years and risk difference between observed and expected joint association														
	HTN		T2DM		CVD		HTN & T2DM		HTN & CVD		T2DM & CVD		HTN, T2DM, & CVD	
	Person‐years at risk (*n* cases)	IR per 1000 person‐years (95% CI)	Person‐years at risk (*n* cases)	IR per 1000 person‐years (95% CI)	Person‐years at risk (*n* cases)	IR per 1000 person‐years (95% CI)	Person‐years at risk (*n* cases)	IR per 1000 person‐years (95% CI)	Person‐years at risk (*n* cases)	IR per 1000 person‐years (95% CI)	Person‐years at risk (*n* cases)	IR per 1000 person‐years (95% CI)	Person‐years at risk (*n* cases)	IR per 1000 person‐years (95% CI)
BMI < 25 kg/m^2^, *“healthy”*	4,474,483 (12,861)	2.87 (2.82–2.92)	4,474,483 (12,861)	2.87 (2.82–2.92)	4,474,483 (12,861)	2.87 (2.82–2.92)	4,474,483 (12,861)	2.87 (2.82–2.92)	4,474,483 (12,861)	2.87 (2.82–2.92)	4,474,483 (12,861)	2.87 (2.82–2.92)	4,474,483 (12,861)	2.87 (2.82–2.92)
BMI ≥ 25 kg/m2, *“healthy”*	7,980,068 (25,221)	3.16 (3.12–3.20)	7,980,068 (25,221)	3.16 (3.12–3.20)	7,980,068 (25,221)	3.16 (3.12–3.20)	7,980,068 (25,221)	3.16 (3.12–3.20)	7,980,068 (25,221)	3.16 (3.12–3.20)	7,980,068 (25,221)	3.16 (3.12–3.20)	7,980,068 (25,221)	3.16 (3.12–3.20)
BMI < 25 kg/m^2^, with condition	297,165 (1544)	5.20 (4.94–5.46)	38,861 (262)	6.74 (5.95–7.61)	51,488 (310)	6.02 (5.37–6.73)	16,386 (132)	8.06 (6.74–9.55)	19,692 (116)	5.89 (4.87–7.07)	3014 (20)	6.64 (4.05–10.25)	2750 (17)	6.18 (3.60–9.90)
BMI ≥ 25 kg/m2, with condition (joint effect)	1,016,610 (5272)	5.19 (5.05–5.33)	204,553 (1257)	6.15 (5.81–6.49)	121,377 (830)	6.84 (6.38–7.32)	128,575 (888)	6.91 (6.46–7.38)	61,190 (381)	6.23 (5.62–6.88)	13,737 (104)	7.57 (6.19–9.17)	14,461 (97)	6.71 (5.44–8.18)
Difference between observed and expected joint effect^a^		−0.3 (−0.41, −0.19)		−0.88 (−1.40, −0.44)		0.53 (0.31, 0.71)		−1.44 (−2.45, −0.58)		0.05 (−0.47, 0.45)		0.64 (−1.36, 1.84)		0.24 (−2.00, 1.54)

*Note*: The model was adjusted by sex, the geographic region of nationality, the MEDEA deprivation index, smoking status, alcohol intake, and stratified by age (5‐year categories). A RERI of 0 denotes lack of additive interaction.

Abbreviations: BMI, Body mass index; CI, confidence interval; CVD, cardiovascular disease; HR, hazard ratio; HTN, hypertension; RERI, relative excess risk due to interaction; T2DM, type 2 diabetes mellitus.

^a^ This was calculated by subtracting the observed joint effect (risk of obesity‐related cancer among people with BMI ≥25 kg/m2 and the condition) and the expected joint effect (risk of obesity‐related cancer among people with BMI ≥25 kg/m2 and “healthy” + people with BMI < 25 kg/m^2^ and the condition—people with BMI < 25 kg/m^2^ and “healthy”).

The corresponding IR per 1000 person‐years and the risk difference between observed and expected joint association are shown in Table 2B. The IR of obesity‐related cancers among participants without a cardiometabolic condition (the *“healthy”*) was 2.87 (95% CI: 2.82–2.92) per 1000 person‐years among those with a BMI < 25 kg/m^2^ and 3.16 (3.12–3.20) among those with a BMI≥25 kg/m^2^ (Table 2B). Analogous to the RERI, the observed joint association of overweight/obesity and CVD led to 53 (95% CI: 31–71) more cases of obesity‐related cancers per 100,000 person‐years than expected. In contrast, the observed joint association of overweight/obesity and HTN led to 30 (95% CI: 19–41) and that of T2DM to 88 (44–140) fewer cases of obesity‐related cancers per 100,000 person‐years than expected (Table 2B).

## 4 DISCUSSION

In this cohort study of 1,774,904 adults in Catalonia, we found that a higher BMI increased the risk of obesity‐related cancer similarly among individuals free of cardiometabolic conditions and those with *HTN*. We also found that the joint association of overweight/obesity (BMI≥25 kg/m^2^) and *CVD* was 0.19 times larger than the sum of the separate associations. This translated into 53 (31 to 71) additional obesity‐related cancer cases per 100,000 person‐years among adults with a BMI≥25 kg/m^2^ and *CVD* as compared to those with a BMI≥25 kg/m^2^ without CVD. If confirmed in subsequent studies, these findings are important to guide public health interventions for overweight/obesity prevention because population subgroups that are jointly exposed to a BMI≥25 kg/m^2^ and a *CVD* would obtain a greater risk reduction of obesity‐related cancer than would others. In contrast, among individuals with *T2DM*, a higher BMI was not associated with an increased risk of obesity‐related cancer. The joint association of overweight/obesity and *T2DM* was associated with fewer than expected obesity‐related cancer cases (−88, −140 to −44 per 100,000 person‐years). However, the IR among those with *T2DM* was still more than double than that of those without overweight/obesity and *T2DM*.

The positive association between BMI and obesity‐related cancers among “*healthy”* individuals is in line with well‐established evidence.^2^, ^10^, ^11^, ^12^ Three mechanisms by which higher general adiposity can increase cancer risk have been extensively reported in the literature: sex hormonal metabolism, insulin and insulin‐like growth factors (IGF) signaling, and adipokine pathways.^28^, ^29^, ^30^, ^31^, ^32^ It has also been suggested that other factors, such as cardiometabolic conditions, could be mediators in the association between body fatness and cancer risk.^7^, ^28^, ^29^ However, since the “*healthy”* population did not include individuals with *HTN*, *T2DM*, and *CVD* by definition, our results support the existence of pathways between body fatness and cancer risk independent of these conditions.

Our results revealed that the BMI‐obesity‐related cancer association still remains present among individuals with an incident diagnosis of *HTN*. This observation could be explained by an independent (from *HTN*) pathway between BMI and cancer risk. Mechanistic pathways that are implicated in the pathogenesis of HTN, T2DM, or CVD—such as chronic inflammation—can also drive cancer progression. However, for HTN and CVD, independent mechanisms of those from obesity have been proposed. For example, hypoxia due to HTN may promote cancer development in the kidneys,^33^ while extracellular matrix proteins secreted from the remodeled heart may promote cancer in the breast.^34^ In contrast, pathways that have been proposed to explain T2DM‐cancer associations (hyperinsulinemia, hyperglycemia, IGF signaling, and inflammation) have also been proposed as possible mediators for the BMI‐cancer relationship.^7^, ^35^, ^36^, ^37^, ^38^, ^39^ The absence of an association between BMI and obesity‐related cancers among people with T2DM could thus be explained by biological pathways that are largely shared between adiposity and T2DM.

This study has several strengths. The size of SIDIAP offered the possibility to reliably address our research question, whereas traditional cohort studies may often lack statistical power to detect interactions and investigate the co‐occurrence of incident morbidities longitudinally. Furthermore, individuals included in SIDIAP are representative of the general population living in Catalonia in terms of age, sex, and geographic distribution which supports the external validity of these findings.^14^ We implemented an advanced multiple imputation methodology to include the individuals eligible to enter the study (with and without a BMI assessment at baseline) and to update their BMI levels during follow‐up, minimizing the possibility of selection bias and exposure misclassification, respectively.^40^ Cancer diagnoses registered in SIDIAP have been validated using these cancer registries thanks to this linkage and priorly used for epidemiological research.^12^, ^17^, ^40^, ^41^

Our findings should be interpreted in light of some limitations. First, data missingness was an important constraint regarding our exposure assessment given that there was a high proportion of individuals who did not have a BMI assessment at baseline. We pre‐specified in our statistical analysis plan that we would deal with missing data using multiple imputations as this method accounts for the uncertainty associated with missing data. We used information from any recording in the individuals' health records (also during follow‐up) for the time‐raster multiple imputations; however, 28% of the study participants did not have any BMI assessment which likely introduced high variability in their BMI estimations among the imputed datasets. Further, the decision to assess BMI in primary care could be related to the patient's apparent weight or their health status. Nevertheless, we already reported in the methods section that the distribution of BMI in SIDIAP was similar to population‐based surveys of the Spanish population suggesting that those with BMI information in SIDIAP were not substantially different in terms of BMI to the broader population.^12^ Our sensitivity analyses, restricted to individuals with a baseline BMI or any real BMI assessment, provided additional support, as the results remained consistent with the main analysis results. Second, we did not have enough statistical power to look at specific cancer types as separate outcomes because the number of at‐risk individuals was modest. Most obesity‐related cancers were cases of the breast (postmenopausal) (34%) and colorectum (32%), therefore results could be driven mainly by these two cancer types. Nevertheless, when we explored associations for other specific cancer types in secondary analyses (for single cardiometabolic conditions or also for HTN & CVD or HTN & T2DM for the most frequent cancer types), the associations were consistent (although with wider CIs) with those of the obesity‐related cancers combined. Third, we used incident cancer diagnoses registered in primary care and hospital records to define cancer cases, which may be prone to outcome misclassification. Although we previously compared cancer cases registered in our data source to those of two provincial population‐based cancer registries and found that the sensitivities of obesity‐related cancers were largely acceptable ranging between 75% and 90% (except for gallbladder and biliary tract cancers that had a sensitivity of 50%), we also observed that SIDIAP includes a considerable number of cases that were not in the registries (positive predictive values for the obesity‐related cancer types included in this study ranged from 47.7 (42.8–52.6) for ovary cancer to 70.1 (68.7–71.5) for colorectal cancer).^17^ Outcome misclassification could have biased our results from the main and secondary analysis towards the null (if a true association between BMI and obesity‐related cancer risk exists) because modest positive predictive values have been reported in the above‐mentioned validation study of SIDIAP cancer diagnoses.^17^ Further limitations included a lack of information about the histological subtypes of cancers (e.g., to define esophageal adenocarcinoma and renal cell cancer). Also, the use of EHRs as the data source of this study limited our analyses in terms of covariate data availability. We were thus not able to adjust for potential confounders such as aspirin use, family history of cancer, hormone therapy among women, and more precisely for diet or physical activity. While we cannot discard that some of the observed associations could be confounded by these factors, we were reassured by the fact that traditional cohort studies (e.g., European Prospective Investigation into Cancer and Nutrition or National Health and Nutrition Examination Survey studies) investigating the association between BMI and risk of specific cancer types with data on diet and physical activity found similar results to ours.^42^, ^43^ For socioeconomic status, we only had data on the MEDEA deprivation index, an ecological indicator of deprivation, therefore there could have also been residual confounding (Figure S1). Despite adjustment for and imputation of missing data on smoking status, residual confounding cannot be excluded given the difficulty of capturing smoking in primary care settings.^44^ However, our analysis restricted to never smokers suggested that such a bias may not have been substantial. Finally, we cannot discard the possibility of collider bias whereby stratification on cardiometabolic conditions could distort associations between BMI and obesity‐related cancer risk.^45^ Although our sensitivity analyses largely supported our main findings, in particular for the sub‐group with CVD, we found some indication of a collider bias among sub‐groups with T2DM alone or combined with HTN. In these two sub‐groups, associations between BMI and our negative control outcome (non‐obesity‐related cancers combined), were inverse and differed from the “healthy” (Tables S6 and S9). Therefore, the findings observed for individuals with these conditions should be interpreted with caution and further investigated in future research.

In this large Southern European study, we found that a higher BMI increased the risk of obesity‐related cancers similarly among adults free of cardiometabolic conditions and among those with HTN, but not among adults with T2DM. Furthermore, individuals with both overweight/obesity and CVD accounted for the highest number of obesity‐related cancer cases compared to those with other individual or combined cardiometabolic conditions. Our findings reinforce the need for public health strategies focusing on the reduction of overweight and obesity for cancer prevention among the general population, but also among population groups with HTN or CVD.

## AUTHOR CONTRIBUTIONS

**Martina Recalde:** Conceptualization (equal); data curation (equal); formal analysis (equal); writing – original draft (equal). **Andrea Pistillo:** Data curation (equal); formal analysis (supporting); software (equal); writing – review and editing (supporting). **Vivian Viallon:** Formal analysis (supporting); writing – review and editing (supporting). **Emma Fontvieille:** Investigation (supporting); methodology (supporting); writing – review and editing (supporting). **Talita Duarte‐Salles:** Conceptualization (equal); data curation (supporting); funding acquisition (equal); supervision (equal); writing – review and editing (equal). **Heinz Freisling:** Conceptualization (equal); funding acquisition (equal); methodology (equal); supervision (equal); writing – review and editing (equal).

## FUNDING INFORMATION

Funding [grant number: IIG_2019_1978] was obtained from World Cancer Research Fund (UK), as part of the World Cancer Research Fund International grant program. MR was also funded by Wereld Kanker Onderzoek Fonds (WKOF, grant number: 2017/1630), as part of the international grants from the World Cancer Research Fund. TDS acknowledges receiving financial support from the Instituto de Salud Carlos III (ISCIII; Miguel Servet 2021: CP21/00023). The funders had no role in study design, data collection, analysis, decision to publish, or preparation of the manuscript.

## CONFLICT OF INTEREST STATEMENT

The authors declare no conflicts of interest.

## ETHICS STATEMENT

We obtained approval from the Clinical Research Ethics Committee of the IDIAPJGol (project code: 20/237‐P) to perform this study.

## CODE AVAILABILITY

The analytical code used in this study is available upon request.

## DISCLAIMER

Where authors are identified as personnel of the International Agency for Research on Cancer and World Health Organization, the authors alone are responsible for the views expressed in this Article and they do not necessarily represent the decisions, policy, or views of the International Agency for Research on Cancer and World Health Organization.

## Supporting information


Data S1.

## Data Availability

In accordance with current European and national law, the data used in this study is only available for the researchers participating in this study. Thus, we are not allowed to distribute or make publicly available the data to other parties. However, researchers from public institutions can request data from SIDIAP if they comply with certain requirements. Further information is available online (https://www.sidiap.org/index.php/menu‐solicitudesen/application‐proccedure) or by contacting Anna Moleras (amoleras@idiapjgol.org).
