# Supplementary material for: Body mass index and incident cardiometabolic conditions in relation to obesity‐related cancer risk: A population‐based cohort study in Catalonia, Spain
Source: Cancer Med. 2023 Sep 28;12(19):20188–200. doi: 10.1002/cam4.6603 (PMC10587966; doi:10.1002/cam4.6603)
Supplement: Supplementary file 1 — Data S1. [file CAM4-12-20188-s001.docx]

**Supplementary Material**

[Table S1. Diagnostic codes for the definition of cancer, hypertension, type 2 diabetes mellitus, cardiovascular disease cases 2](#_heading=h.30j0zll)

[Appendix 1. Description of the multiple imputations’ methodology for missing data on BMI and the covariates of interest 4](#_heading=h.1fob9te)

[Figure S1. Directed Acyclic Graph for the possible causal effect of obesity on cancer used to adjust the Cox proportional hazard models 8](#_heading=h.3znysh7)

[Table S2. Descriptive statistics of the SIDIAP population excluded at each step of the definition of the study population 9](#_heading=h.2et92p0)

[Table S3. Descriptive statistics of the study population by type of body mass index assessment 11](#_heading=h.tyjcwt)

[Table S4. Descriptive statistics of the study population at the moment of the diagnosis of a cardiometabolic condition (ie, HTN, T2DM, or CVD) or a combination of these conditions 13](#_heading=h.3dy6vkm)

[Figure S2. Association between body mass index and the risk of obesity-related cancers by ascertainment of incident cardiometabolic conditions, with 95% CIs, minimally- and fully-adjusted models 16](#_heading=h.1t3h5sf)

[Appendix 2. Description of the supplementary and sensitivity analyses of the primary objective of this study. 17](#_heading=h.4d34og8)

[Figure S3. Association between body mass index and the risk of obesity-related cancers by ascertainment of incident cardiometabolic conditions, with 95% CIs, stratified by sex and age 19](#_heading=h.2s8eyo1)

[Figure S4. Association between body mass index and the risk of specific cancer types by ascertainment of incident cardiometabolic conditions, with 95% CI 22](#_heading=h.17dp8vu)

[Figure S5. Sensitivity analyses: association between body mass index and the risk of obesity-related cancers by ascertainment of incident cardiometabolic conditions, with 95% CIs 23](#_heading=h.3rdcrjn)

[Table S5. Sensitivity analysis: association between body mass index and the risk of obesity-related cancers by ascertainment of incident cardiometabolic conditions, among never smokers, with 95% CIs 24](#_heading=h.26in1rg)

[Table S6. Sensitivity analysis: association between body mass index and the risk of non-obesity-related cancers by ascertainment of incident cardiometabolic conditions, with 95% Cis 25](#_heading=h.lnxbz9)

[Table S7. Sensitivity analysis: association between body mass index and the risk of obesity-related cancers by ascertainment of incident cardiometabolic conditions, restricting the definition of obesity-related cancers, with 95% CIs 26](#_heading=h.35nkun2)

[Figure S6. Association between incident cardiometabolic conditions and the risk of obesity-related cancers, with 95% CIs 27](#_heading=h.1ksv4uv)

[Table S8. Sensitivity analysis: Relative risk of obesity-related cancers due to interaction between overweight/obesity (BMI≥25 kg/m2) and incident cardiometabolic conditions, among never smokers, with 95% CIs 28](#_heading=h.z337ya)

[Table S9. Sensitivity analysis: Relative risk of non-obesity-related cancers due to interaction between overweight/obesity (BMI≥25 kg/m2) and incident cardiometabolic conditions, with 95% CIs 29](#_heading=h.3j2qqm3)

[Appendix 3. STROBE Statement checklist 30](#_heading=h.4i7ojhp)

#

# Table S1. Diagnostic codes for the definition of cancer, hypertension, type 2 diabetes mellitus, cardiovascular disease cases

| **Outcome** | **ICD-10 codes^1^** | **ICD-9 codes^2^** |
| --- | --- | --- |
| **Obesity-related cancers^3^** |  |  |
| Colorectal | C18-C21 | 153, 154 |
| Liver | C22 | 155 |
| Gallbladder & biliary tract | C23-C24 | 156 |
| Pancreas | C25 | 157 |
| Breast postmenopausal | C50 | 174, 175 |
| Corpus Uteri | C54-C55 | 179, 182 |
| Ovary | C56 | 183, 183.0 |
| Kidney | C64 | 189.0 |
| Brain and CNS^4^ | C70-C72, C75.1-C75.3 | 191, 192, 194.3, 194.4 |
| Thyroid | C73 | 193 |
| Multiple myeloma | C90 | 203 |
| **Non-obesity related cancers^3^** |  |  |
| Esophagus^5^ | C15 | 150 |
| Stomach^5^ | C16 | 151 |
| Head and neck | C00-C14 | 140-149 |
| Larynx | C32 | 161 |
| Trachea, bronchus & Lung | C33-C34 | 162 |
| Bone and articular cartilage | C40-C41 | 170 |
| Malignant melanoma of skin | C43 | 172 |
| Connective and soft tissue | C47, C49 | 171 |
| Breast premenopausal | C50 | 174, 175 |
| Cervix Uteri | C53 | 180 |
| Penis | C60 | 187.1-187.4 |
| Prostate | C61 | 185 |
| Testis | C62 | 186 |
| Urinary Tract | C65, C66, C68 | 189.1-189.9 |
| Bladder | C67 | 188 |
| Hodgkin lymphoma | C81 | 201 |
| Non-Hodgkin Lymphoma | C82-C86, C96 | 200, 202 |
| Leukemia | C91-C95 | 204-208 |
| Others and non-specific | C17, C26, C30, C31, C37-C39, C4A, C45, C46, C48, C51, C52, C57, C58, C63, C69, C74, C75.0, C75.4-C75.9, C7A, C76, C80, C88, C97 | 152, 158-160, 163-165, 176, 181, 184, 187.5-187.9, 190, 194.0, 194.1, 194.5-194.9, 195, 199, 209.1-209.3, 273.3, 279.5 |
| **Hypertension^6^** | I10 | - |
| **Type 2 Diabetes Mellitus^6^** | E11 | - |
| **Cardiovascular disease^7^** |  |  |
| Coronary disease | I20-I25 | 410-414 |
| Cerebrovascular disease | I60-I69 | 430-434, 436-438 |

Notes: 1) ICD-10 is the classification system used in the SIDIAP. 2) ICD-9 is the classification system used in the hospital discharge database. 3) We prioritized the type of cancer and date registered in the SIDIAP over the hospital discharge database. 4) Include pituitary gland and pineal gland tumors. 5) We did not consider these cancers as obesity-related because with the available data we could not differentiate esophageal adenocarcinoma (obesity-related) from squamous cell carcinoma nor gastric cardia (obesity-related) from non-cardia cancers. 6) We only used diagnoses registered in the SIDIAP database. 7) We prioritized the date registered in the hospital discharge database over the SIDIAP.

Abbreviations: CNS: Central Nervous System; ICD-9: International Classification for Diseases, 9th

revision; ICD-10: International Classification for Diseases, 10th revision; SIDIAP: Information System for Research in Primary Care.

# Appendix 1. Description of the multiple imputations’ methodology for missing data on BMI and the covariates of interest

We assumed BMI was missing at random after inspecting the distribution of the characteristics of those with a BMI assessment at baseline, with at least one BMI assessment during the follow-up period, and without any BMI assessment available (Table S3). We applied multilevel time raster multiple imputations to have BMI assessments for all the study participants and to be able to update BMI values every time a participant was diagnosed with a cardiometabolic condition (ie, HTN, T2DM, or CVD) or a combination of these conditions (see definition of cardiometabolic conditions in the “Covariates of interest” section).

Our primary dataset was in long format and contained as many rows per participant as years with available valid BMI values the individual had. To be considered as valid BMIs, the BMI measurements had to be i) comprised between 15kg/m^2^ and 60kg/m^2^ (ie, extremely low values could be indicative of an underlying disease, and extremely low/high values could be due to data entry errors in medical records); ii) measured at least one year before a cancer diagnosis to prevent reverse causality (ie, undiagnosed cancer affecting BMI). If more than one BMI measurement was available per year, we took the nearest value to the mean for that year.

As a first step to applying the multilevel time raster multiple imputations, we set up the time raster. We chose three time-points to impute BMI for each individual: 01/01/2010, 01/01/2013, 01/01/2016. For simplicity, we considered time in years (ie, 2010, 2013, 2016). We added two extra time points (2006 and 2018) as boundaries to consider all BMI values registered in an individual’s electronic health record (2006 was the first year of BMI assessments available in SIDIAP and 2018 was the last year of follow-up in this study). Operationally, one row per time point (eg, 2010) with a missing value for BMI (which was imputed in the second step of this procedure) was added to the primary dataset for each individual. The information on all the other variables (eg, sex, nationality, etc.) was replicated for each of these rows.

We also added 5 columns that represented a B-spline of degree 1 for the 5 time-points of interest. Considering all valid available BMI measurements, we checked if the year of measurement of the real BMI measurement coincided with one of the time points of interest. In such a case, the B-spline variable corresponding to that column received a 1 (eg, if a person had a real BMI measurement in 2010, then that measurement was attributed a weight of 1/1 for the time point of 2010, and 0 for all the other time points). In all other cases, the spline coefficients for time points were distributed over two adjacent columns, that summed 1 (eg, if a person had a real BMI measurement in 2011, then that measurement was attributed a weight of 0.75/1 for the time point of 2010, 0.25/1 for the time point of 2013, and 0 for all the other time points).

As a second step, we set up the imputation models. Firstly, we specified the model (predictive mean matching with 5 imputations) to impute the categorical variables with missing data at baseline (socioeconomic status, smoking status, alcohol intake). The predictor variables were BMI, sex, age at baseline, socioeconomic status, smoking status, alcohol intake, geographic region of nationality, number of visits to primary care centers, the Charlson Comorbidity index, diagnosis of obesity-related cancers, and follow-up time. Secondly, we specified the multilevel model to impute BMI. We used a linear mixed-effects model with PAN implementation, with 5 imputations. The cluster variable was each individual. Level 1 (ie, that can vary within clusters) variables were the year of BMI measurement (B-spline represented) and three distinct indicator variables for which the value was 1 if the individual had been diagnosed with HTN, T2DM, and/or CVD before the BMI assessment, 0 if otherwise (as these conditions can lead to changes in BMI). Level 2 (ie, that vary between clusters) variables were sex, age at baseline, socioeconomic status, smoking status, alcohol intake, geographic region of nationality, number of visits to primary care centers, the Charlson Comorbidity index, diagnosis of obesity-related cancer, and follow-up time. The model, using the same notation as in Woltman et al.^1^, for subject *i* at time *t* was:

$BMI_{ti} = \beta_{0i}+ \beta_{1i}(yea{rBMI}_{ti}+i{HTN}_{ti}+iT2DM_{ti}+iCVD{}_{ti})+e_{ti}$,

$$\beta_{ki} = \gamma_{k0}+\gamma_{k1}(sex_{i}+age_{i}+SES_{i}+smoking_{i}+alcohol_{i}+nationality_{i}+n.visits_{i}+$$

$Charlson_{i}+obesity.cancers_{i}+follow.up_{i})+u_{ki}$,

where $k=\{0,1\}$, $e_{ti}～N(0,\sigma_{t}^{2})$, $u_{ki}～N(0,\sigma_{k}^{2})$.

To implement the multilevel time raster multiple imputations we used the library MICE 3.13.0 available for the software R version 4.0.3.

In Figure 1 we can see the distribution of observed BMI values (in blue) and of those obtained with the multilevel time raster multiple imputations (in red). It can also be observed that some multiply imputed BMI values were outside our pre-established limits for valid BMIs (ie, <15kg/m^2^ and >60kg/m^2^). Thus as post-estimation processing, we assigned values <15kg/m^2^ to 15kg/m^2^ and those >60kg/m^2^ to 60kg/m^2^.


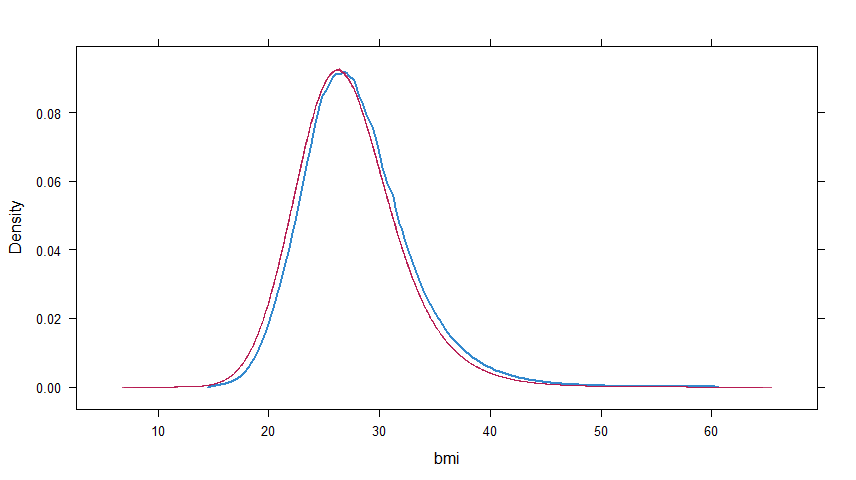


**Figure 1.** Distribution of observed (blue) and multiply imputed (red) BMI values.

In Figure 2, we exemplify the results of the multiple imputations for a random sample of 9 individuals. The x-axis corresponds to the time period of interest and the y-axis to the BMI values. We observed real BMI measurements (black dots) throughout follow-up (x-axis) for each individual (box). BMI trajectories are represented with the blue lines (one per imputation) joining the imputed values at each time point (2006, 2010, 2013, 2016, and 2018) indicated with dotted grey vertical lines, emerging from the x-axis. Vertical colored lines indicate a diagnosis of HTN, T2DM, and/or CVD in the corresponding year.


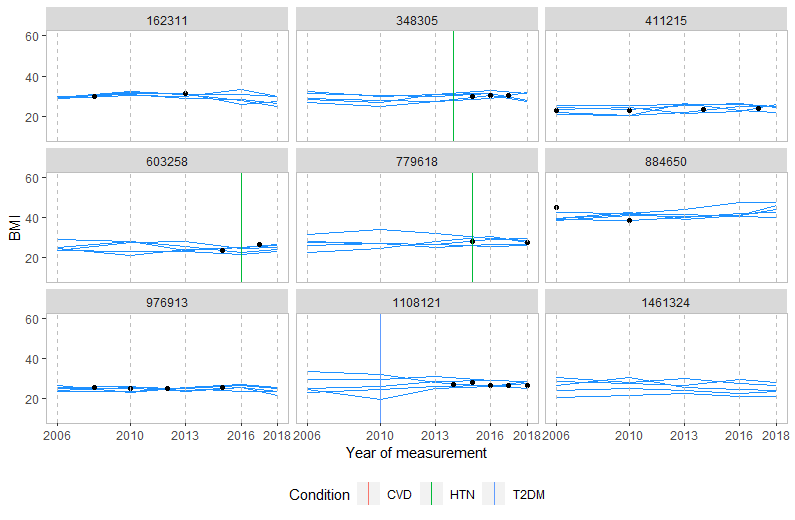
**Figure 2.** Five multiply imputed trajectories of BMI for a random sample of 9 individuals (in blue) with observed BMI measurements (black dots).

**References**

1. Woltman, H., Feldstain, A., MacKay, J. C., & Rocchi, M. (2012). An Introduction to Hierarchical Linear Modeling. Tutorials in Quantitative Methods for Psychology, 8, 52-69.. Quantitative Methods Psychology tutorial. 8. 52-69.

# Figure S1. Directed Acyclic Graph for the possible causal effect of obesity on cancer used to adjust the Cox proportional hazard models

**
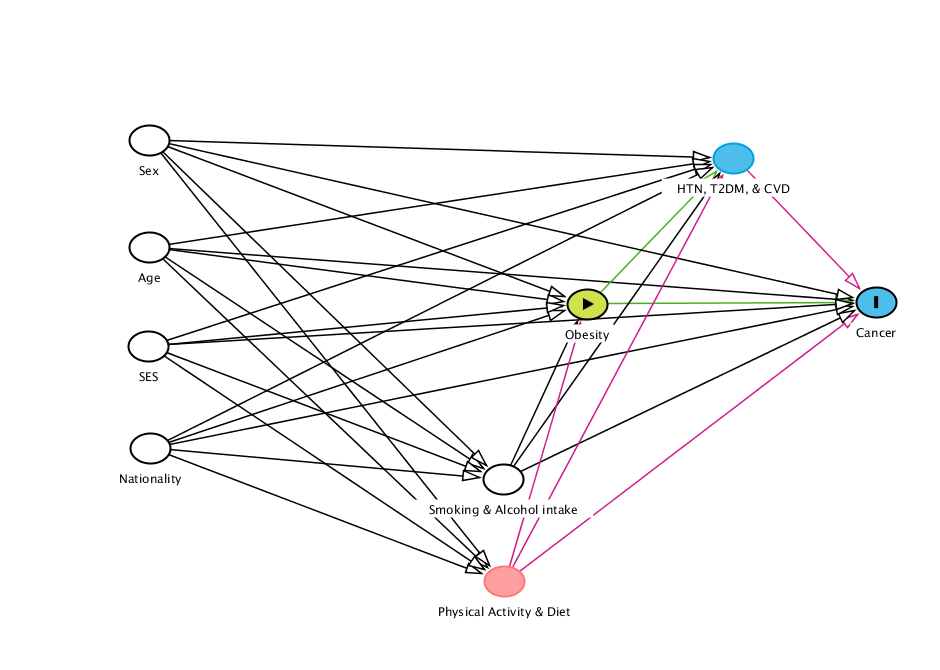
**

Notes: Green arrows indicate a possible pathway between the exposure and outcome of interest. Red arrows indicate possible confounding factors modifying the association between the exposure and outcome of interest that cannot be accounted for with the available data.

Abbreviations: CVD: Cardiovascular disease; HTN: Hypertension; SES: socioeconomic status; T2DM: Type 2 diabetes mellitus.

# Table S2. Descriptive statistics of the SIDIAP population excluded at each step of the definition of the study population

|  | **Initial population** | **With at least 1 year of prior history** | **Without history of cancer** | **Without history of**  **HTN** | **Without history of T2DM** | **Without history of CVD** | **With at least 1 year of follow-up** |
| --- | --- | --- | --- | --- | --- | --- | --- |
| **N** | 3,097,073 | 2,952,301 | 2,790,169 | 1,957,202 | 1,858,870 | 1,819,070 | 1,774,904 |
| **BMI in kg/m^2^, median (IQR)** | 28 (24.8, 30.9) | 28 (24.8, 30.9) | 28 (24.8, 30.9) | 27 (24.1, 29.9) | 27 (24.0, 29.8) | 27 (24.0, 29.7) | 27 (24.0, 29.8) |
| **Age in years, median (IQR)** | 57 (47.0, 69.0) | 57 (47.0, 70.0) | 56 (47.0, 69.0) | 52 (45.0, 62.0) | 51 (45.0, 61.0) | 51 (45.0, 61.0) | 51 (44.0, 60.0) |
| **Female sex, n (%)** | 1,629,961 (52.6) | 1,553,806 (52.6) | 1,472,481 (52.8) | 1,004,486 (51.3) | 966,400 (52.0) | 952,313 (52.4) | 931,239 (52.5) |
| **Nationality, n (%)** |  |  |  |  |  |  |  |
| Spanish | 2,843,846 (91.8) | 2,765,577 (93.7) | 2,606,573 (93.4) | 1,795,523 (91.7) | 1,702,404 (91.6) | 1,663,886 (91.5) | 1,663,886 (91.5) |
| Global North | 86,887 (2.8) | 63,227 (2.1) | 61,729 (2.2) | 53,422 (2.7) | 52,477 (2.8) | 51,866 (2.9) | 48,735 (2.7) |
| Global South | 166,340 (5.4) | 123,497 (4.2) | 121,867 (4.4) | 108,257 (5.5) | 103,989 (5.6) | 103,318 (5.7) | 93,530 (5.3) |
| **MEDEA deprivation index, n (%)** |  |  |  |  |  |  |  |
| Quintile 1 (least deprived) | 493,585 (15.9) | 464,984 (15.7) | 439,070 (15.7) | 323,143 (16.5) | 311,345 (16.7) | 305,062 (16.8) | 298,244 (16.8) |
| Quintile 2 | 442,201 (14.3) | 423,668 (14.4) | 401,257 (14.4) | 287,500 (14.7) | 274,421 (14.8) | 268,996 (14.8) | 263,369 (14.8) |
| Quintile 3 | 429,009 (13.9) | 410,582 (13.9) | 389,321 (14.0) | 273,343 (14.0) | 259,621 (14.0) | 254,467 (14.0) | 249,361 (14.0) |
| Quintile 4 | 415,714 (13.4) | 396,473 (13.4) | 376,069 (13.5) | 261,214 (13.3) | 246,564 (13.3) | 241,493 (13.3) | 236,343 (13.3) |
| Quintile 5 (most deprived) | 379,979 (12.3) | 358,176 (12.1) | 340,680 (12.2) | 236,981 (12.1) | 222,524 (12.0) | 218,071 (12.0) | 211,990 (11.9) |
| Rural | 573,466 (18.5) | 554,961 (18.8) | 525,887 (18.8) | 361,501 (18.5) | 342,955 (18.4) | 335,555 (18.4) | 328,793 (18.5) |
| Missing | 363,119 (11.7) | 343,457 (11.6) | 317,885 (11.4) | 213,520 (10.9) | 201,440 (10.8) | 195,426 (10.7) | 186,804 (10.5) |
| **Smoking status, n (%)** |  |  |  |  |  |  |  |
| Never smoker | 1,126,426 (36.4) | 1,114,946 (37.8) | 1,041,039 (37.3) | 612,819 (31.3) | 569,794 (30.7) | 554,424 (30.5) | 544,509 (30.7) |
| Former smoker | 221,001 (7.1) | 220,024 (7.5) | 201,601 (7.2) | 118,586 (6.1) | 105,559 (5.7) | 100,237 (5.5) | 97,677 (5.5) |
| Current smoker | 310,932 (10.0) | 307,114 (10.4) | 293,627 (10.5) | 230,680 (11.8) | 218,793 (11.8) | 215,064 (11.8) | 211,160 (11.9) |
| Missing | 1,438,714 (46.5) | 1,310,217 (44.4) | 1,253,902 (44.9) | 995,117 (50.8) | 964,724 (51.9) | 949,345 (52.2) | 921,558 (51.9) |
| **Alcohol intake, n (%)** |  |  |  |  |  |  |  |
| No risk | 1,089,894 (35.2) | 1,079,844 (36.6) | 1,002,068 (35.9) | 559,927 (28.6) | 510,185 (27.4) | 493,668 (27.1) | 484,601 (27.3) |
| Low risk | 483,210 (15.6) | 479,474 (16.2) | 448,665 (16.1) | 272,960 (13.9) | 250,061 (13.5) | 243,164 (13.4) | 239,641 (13.5) |
| High risk | 49,951 (1.6) | 49,497 (1.7) | 46,872 (1.7) | 31,141 (1.6) | 28,722 (1.5) | 28,053 (1.5) | 27,491 (1.5) |
| Missing | 1,474,018 (47.6) | 1,343,486 (45.5) | 1,292,564 (46.3) | 1,093,174 (55.9) | 1,069,902 (57.6) | 1,054,185 (58.0) | 1,023,171 (57.6) |
| **Charlson comorbidity index, n (%)** |  |  |  |  |  |  |  |
| 0 | 2,093,450 (67.6) | 1,963,820 (66.5) | 1,957,745 (70.2) | 1,561,867 (79.8) | 1,560,729 (84.0) | 1,549,893 (85.2) | 1,522,931 (85.8) |
| 1 | 552,817 (17.8) | 542,903 (18.4) | 540,747 (19.4) | 293,709 (15.0) | 231,974 (12.5) | 214,758 (11.8) | 210,730 (11.9) |
| 2 | 263,230 (8.5) | 259,442 (8.8) | 182,626 (6.5) | 71,944 (3.7) | 48,988 (2.6) | 41,742 (2.3) | 31,995 (1.8) |
| ≥ 3 | 187,576 (6.1) | 186,136 (6.3) | 109,051 (3.9) | 29,682 (1.5) | 17,179 (0.9) | 12,677 (0.7) | 9,248 (0.5) |

Notes: 1) The statistics of BMI, the MEDEA deprivation index, smoking status, and alcohol intake were calculated using the complete-case approach.

Abbreviations: BMI: Body Mass Index; CVD: Cardiovascular disease; HTN: Hypertension; IQR: Interquartile range; MEDEA: “Mortalidad en áreas pequeñas Españolas y Desigualdades Socioeconómicas y Ambientales”; SIDIAP: Information System for Research in Primary Care; T2DM: Type 2 diabetes mellitus.

# Table S3. Descriptive statistics of the study population by type of body mass index assessment

|  | **All study participants** | | **All study participants by type of BMI assessment** | | |
| --- | --- | --- | --- | --- | --- |
|  | **Imputed data for BMI and covariates^1^** | **With missing information^2^** | **At baseline** | **Over follow-up** | **Never** |
| **N** | 1,774,904 | 1,774,904 | 681,386 | 589,319 | 504,199 |
| **Follow-up time in years, median (IQR)** | 8.0 (8.0, 8.0) | 8.0 (8.0, 8.0) | 8.0 (8.0, 8.0) | 8.0 (8.0, 8.0) | 8.0 (8.0, 8.0) |
| **N of visits to primary care centers, median (IQR)** | 3.0 (0.0, 7.0) | 3.0 (0.0, 7.0) | 6.0 (2.0, 10.0) | 2.0 (0.0, 6.0) | 0.0 (0.0, 2.0) |
| **BMI in kg/m^2^, median (IQR)** | 27.0  (23.9, 30.0) | 27.0  (23.9, 29.6) | 27.0 (23.9, 29.6) | 27.0 (24.0, 29.7) | - |
| **Age in years, median (IQR)** | 51.0 (44.0,  60.0) | 51.0 (44.0, 60.0) | 53.0 (45.0, 63.0) | 51.0 (45.0, 59.0) | 49.0 (43.0, 58.0) |
| **Female sex, n (%)** | 931,239  (52.5) | 931,239  (52.5) | 392,323  (57.6) | 300,885 (51.1) | 238,031 (47.2) |
| **Nationality, n (%)** |  |  |  |  |  |
| Spanish | 1,632,639 (92.0) | 1,632,639  (92.0) | 635,444  (93.3) | 549,756 (93.3) | 447,439 (88.7) |
| Global North | 48,735 (2.7) | 48,735 (2.7) | 12,668 (1.9) | 14,058 (2.4) | 22,009 (4.4) |
| Global South | 93,530 (5.3) | 93,530 (5.3) | 33,274 (4.9) | 25,505 (4.3) | 34,751 (6.9) |
| **MEDEA deprivation index, n (%)** |  |  |  |  |  |
| Quintile 1 (least deprived) | 334,723  (18.9) | 298,244  (16.8) | 95,863  (14.1) | 101,905 (17.3) | 100,476 (19.9) |
| Quintile 2 | 294,506  (16.6) | 263,369  (14.8) | 99,017  (14.5) | 91,480  (15.5) | 72,872  (14.5) |
| Quintile 3 | 278,367  (15.7) | 249,361  (14.0) | 99,079  (14.5) | 86,804  (14.7) | 63,478  (12.6) |
| Quintile 4 | 263,856  (14.9) | 236,343  (13.3) | 97,405  (14.3) | 82,192  (13.9) | 56,746  (11.3) |
| Quintile 5 (most deprived) | 236,249  (13.3) | 211,990  (11.9) | 89,335  (13.1) | 72,276  (12.3) | 50,379  (10.0) |
| Rural | 367,203  (20.7) | 328,793  (18.5) | 140,956  (20.7) | 108,718 (18.4) | 79,119  (15.7) |
| Missing | - | 186,804  (10.5) | 59,731  (8.8) | 45,944  (7.8) | 81,129  (16.1) |
| **Smoking status, n (%)** |  |  |  |  |  |
| Never smoker | 1,090,923 (61.5) | 544,509  (30.7) | 323,474  (47.5) | 154,567 (26.2) | 66,468  (13.2) |
| Former smoker | 205,295  (11.6) | 97,677  (5.5) | 60,841  (8.9) | 26,860  (4.6) | 9,976  (2.0) |
| Current smoker | 478,686  (27.0) | 11,160  (11.9) | 108,993  (16.0) | 66,615  (11.3) | 35,552  (7.1) |
| Missing | - | 921,558  (51.9) | 188,078  (27.6) | 341,277 (57.9) | 392,203 (77.8) |
| **Alcohol intake, n (%)** |  |  |  |  |  |
| No risk | 1,099,308 (61.9) | 484,601  (27.3) | 304,768  (44.7) | 130,497 (22.1) | 49,336  (9.8) |
| Low risk | 602,673  (34.0) | 239,641  (13.5) | 150,867  (22.1) | 67,636  (11.5) | 21,138  (4.2) |
| High risk | 72,923  (4.1) | 27,491  (1.5) | 16,296  (2.4) | 7,966  (1.4) | 3,229  (0.6) |
| Missing | - | 1,023,171  (57.6) | 209,455  (30.7) | 383,220 (65.0) | 430,496 (85.4) |
| **Charlson comorbidity index, n (%)** |  |  |  |  |  |
| 0 | 1,522,931 (85.8) | 1,522,931  (85.8) | 545,811  (80.1) | 513,435 (87.1) | 463,685 (92.0) |
| 1 | 210,730 (11.9) | 210,730 (11.9) | 111,738 (16.4) | 65,524 (11.1) | 33,468 (6.6) |
| 2 | 31,995 (1.8) | 31,995 (1.8) | 18,378 (2.7) | 8,315 (1.4) | 5,302 (1.1) |
| ≥ 3 | 9,248 (0.5) | 9,248 (0.5) | 5,459 (0.8) | 2,045 (0.3) | 1,744 (0.3) |
| **Cause of exit from the study, n (%)** |  |  |  |  |  |
| End of study | 1,373,650 (77.4) | 1,373,650  (77.4) | 550,755  (80.8) | 522,514 (88.7) | 300,381 (59.6) |
| Transferred out of the SIDIAP | 219,024  (12.3) | 219,024  (12.3) | 55,798  (8.2) | 32,049  (5.4) | 131,177 (26.0) |
| Death | 78,456 (4.4) | 78,456 (4.4) | 29,517 (4.3) | 14,868 (2.5) | 34,071 (6.8) |
| Obesity related cancers | 49,312 (2.8) | 49,312 (2.8) | 21,694 (3.2) | 9,451 (1.6) | 18,167 (3.6) |
| Non-obesity related cancers | 54,462 (3.1) | 54,462 (3.1) | 23,622 (3.5) | 10,437 (1.8) | 20,403 (4.0) |

Notes: The first two columns should (by design) have the same values for variables without missing data such as age and nationality, and only vary for BMI, MEDEA deprivation index, smoking status and alcohol intake, since both columns have the same underlying population. However, columns 3, 4, and 5, are mutually exclusive and can have different distributions for variables with or without missing data. 1)​​ The statistics of BMI, the MEDEA deprivation index, smoking status, and alcohol intake were calculated using the multiple imputation approach, with 5 data sets created. For visualization purposes, we divided the n for the categorical variables by 5. 2) The median and IQR of the BMI variable for this column corresponds to the 681,386 individuals with a BMI assessment at baseline. Non-obesity related cancers do not include non-melanoma skin cancer.

Abbreviations: BMI: Body Mass Index; IQR: Interquartile range; MEDEA: “Mortalidad en áreas pequeñas Españolas y Desigualdades Socioeconómicas y Ambientales”; SIDIAP: Information System for Research in Primary Care.

# Table S4. Descriptive statistics of the study population at the moment of the diagnosis of a cardiometabolic condition (ie, HTN, T2DM, or CVD) or a combination of these conditions

|  | **General population (healthy)** | **HTN** | **T2DM** | **CVD** | **HTN & T2DM** | **HTN &**  **CVD** | **T2DM & CVD** | **HTN, T2DM, & CVD** |
| --- | --- | --- | --- | --- | --- | --- | --- | --- |
| **N** | 1,774,904 | 296,445 | 65,000 | 56,573 | 39,143 | 26,139 | 6,297 | 6,069 |
| **Follow-up time in years (whole study), median (IQR)** | 8.0 (8.0, 8.0) | 8.0 (8.0, 8.0) | 8.0 (8.0, 8.0) | 8.0 (7.7, 8.0) | 8.0 (8.0, 8.0) | 8.0 (8.0, 8.0) | 8.0 (7.7, 8.0) | 8.0 (7.9, 8.0) |
| **Follow-up time in years (until next state), median (IQR)** | 8.0 (8.0, 8.0) | 5.0 (2.8, 6.9) | 5.0 (2.6, 6.9) | 4.0 (1.5, 6.1) | 4.0 (1.9, 5.7) | 3.0 (1.3, 5.0) | 3.0 (1.2, 4.8) | 2.0 (1.1, 4.3) |
| **N visits to primary care centers, median (IQR)** | 3.0 (0.0, 7.0) | 6.0 (3.0, 10.0) | 7.0 (3.0, 11.0) | 7.0 (3.0, 12.0) | 8.0 (4.0, 12.0) | 8.0 (4.0, 13.0) | 9.0 (5.0, 15.0) | 9.0 (5.0, 15.0) |
| **BMI in kg/m^2^, median (IQR)^1^** | 27.0  (23.9, 30.0) | 28.0  (25.3, 31.4) | 29.0  (26.3, 32.8) | 27.0  (24.4, 30.2) | 30.0  (27.2, 33.8) | 28.0  (25.0, 30.9) | 29.0  (25.8, 31.8) | 29.0  (26.2, 32.5) |
| **Age in years, median (IQR)** | 51.0  (44.0, 60.0) | 60.0  (52.0, 69.0) | 59.0  (52.0, 67.0) | 66.0  (56.0, 78.0) | 62.0  (55.0, 70.0) | 69.0  (59.0, 80.0) | 66.0  (58.0, 76.0) | 68.0  (59.0, 78.0) |
| **Female sex, n (%)** | 931,239 (52.5) | 150,276 (50.7) | 25,683 (39.5) | 20,964 (37.1) | 15,941 (40.7) | 10,469 (40.1) | 1,679 (26.7) | 1,926 (31.7) |
| **Nationality, n (%)** |  |  |  |  |  |  |  |  |
| Spanish | 1,632,639 (92.0) | 281,893 (95.1) | 59,933 (92.2) | 54,631 (96.6) | 36,752 (93.9) | 25,193 (96.4) | 6,050 (96.1) | 5,800 (95.6) |
| Global North | 48,735 (2.7) | 6,033 (2.0) | 942 (1.4) | 876 (1.5) | 768 (2.0) | 478 (1.8) | 81 (1.3) | 115 (1.9) |
| Global South | 93,530 (5.3) | 8,519 (2.9) | 4,125 (6.3) | 1,066 (1.9) | 1,623 (4.1) | 468 (1.8) | 166 (2.6) | 154 (2.5) |
| **MEDEA deprivation index, n (%)^1^** |  |  |  |  |  |  |  |  |
| Quintile 1 (least deprived) | 334,723 (18.9) | 51,725 (17.4) | 9103 (14.0) | 10,059 (17.8) | 5813 (14.8) | 4876 (18.7) | 960 (15.2) | 988 (16.3) |
| Quintile 2 | 294,506 (16.6) | 48,847 (16.5) | 10,077 (15.5) | 9233 (16.3) | 6161 (15.7) | 4238 (16.2) | 940 (14.9) | 978 (16.1) |
| Quintile 3 | 278,367 (15.7) | 48,192 (16.3) | 10,565 (16.3) | 8662 (15.3) | 6490 (16.6) | 4154 (15.9) | 983 (15.6) | 1021 (16.8) |
| Quintile 4 | 263,856 (14.9) | 45,430 (15.3) | 10,916 (16.8) | 8522 (15.1) | 6382 (16.3) | 4015 (15.4) | 1096 (17.4) | 960 (15.8) |
| Quintile 5 (most deprived) | 236,249 (13.3) | 40,379 (13.6) | 11,074 (17.0) | 7663 (13.5) | 6241 (15.9) | 3520 (13.5) | 1006 (16.0) | 925 (15.2) |
| Rural | 367,203 (20.7) | 61,872 (20.9) | 13,265 (20.4) | 12,434 (22.0) | 8056 (20.6) | 5336 (20.4) | 1312 (20.8) | 1197 (19.7) |
| **Smoking status, n (%)^1^** |  |  |  |  |  |  |  |  |
| Never smoker | 1,090,923 (61.5) | 194,626 (65.7) | 39,700 (61.1) | 36,317 (64.2) | 24,437 (62.4) | 17,237 (65.9) | 3776 (60.0) | 3780 (62.3) |
| Former smoker | 205,295 (11.6) | 34,811 (11.7) | 8663 (13.3) | 7482 (13.2) | 5207 (13.3) | 3345 (12.8) | 939 (14.9) | 845 (13.9) |
| Current smoker | 478,686 (27.0) | 67,008 (22.6) | 16637 (25.6) | 12,774 (22.6) | 9499 (24.3) | 5557 (21.3) | 1582 (25.1) | 1444 (23.8) |
| **Alcohol intake, n (%)^1^** |  |  |  |  |  |  |  |  |
| No risk | 1,099,308 (61.9) | 181,053 (61.1) | 38,785 (59.7) | 34,088 (60.3) | 23,035 (58.8) | 15,816 (60.5) | 3594 (57.1) | 3508(57.8) |
| Low risk | 602,673 (34.0) | 102,746 (34.7) | 22,954 (35.3) | 19,909 (35.2) | 14,090 (36.0) | 9087 (34.8) | 2384 (37.9) | 2227 (36.7) |
| High risk | 72,923 (4.1) | 12,646 (4.3) | 3261 (5.0) | 2576 (4.6) | 2018 (5.2) | 1236 (4.7) | 319 (5.1) | 334 (5.5) |
| **Charlson comorbidity index, n (%)** |  |  |  |  |  |  |  |  |
| 0 | 1,522,931 (85.8) | 202,805 (68.4) | 17,850 (27.5) | 20,758 (36.7) | 8,212 (21.0) | 8,084 (30.9) | 598 (9.5) | 447 (7.4) |
| 1 | 210,730 (11.9) | 64,508 (21.8) | 33,295 (51.2) | 21,084 (37.3) | 20,517 (52.4) | 10,081 (38.6) | 2,347 (37.3) | 2,188 (36.1) |
| 2 | 31,995 (1.8) | 19,783 (6.7) | 9,299 (14.3) | 8,673 (15.3) | 6,630 (16.9) | 4,536 (17.4) | 1,856 (29.5) | 1,808 (29.8) |
| ≥ 3 | 9,248 (0.5) | 9,137 (3.1) | 4,538 (7.0) | 6,045 (10.7) | 3,779 (9.7) | 3,433 (13.1) | 1,496 (23.8) | 1,626 (26.8) |
| **Cause of exit from the study, n (%)** |  |  |  |  |  |  |  |  |
| End of study | 1,373,650 (77.4) | 253,271 (85.4) | 54,048 (83.2) | 38,301 (67.7) | 33,176 (84.8) | 19,556 (74.8) | 4,640 (73.7) | 4,510 (74.3) |
| Transferred out of the SIDIAP | 219,024 (12.3) | 14,318 (4.8) | 3,388 (5.2) | 4,167 (7.4) | 1,712 (4.4) | 1,503 (5.8) | 349 (5.5) | 363 (6.0) |
| Death | 78,456 (4.4) | 12,238 (4.1) | 3,274 (5.0) | 10,117 (17.9) | 1,974 (5.0) | 3,619 (13.8) | 876 (13.9) | 890 (14.7) |
| Obesity related cancers | 49,312 (2.8) | 7,607 (2.6) | 1,956 (3.0) | 1,429 (2.5) | 1,079 (2.8) | 516 (2.0) | 141 (2.2) | 114 (1.9) |
| Non-obesity related cancers | 54,462 (3.1) | 9,011 (3.0) | 2,334 (3.6) | 2,559 (4.5) | 1,202 (3.1) | 945 (3.6) | 291 (4.6) | 192 (3.2) |

Notes: 1)​​ The statistics of BMI, the MEDEA deprivation index, smoking status, and alcohol intake were calculated using the multiple imputation approach, with 5 data sets created. For visualization purposes, we divided the n for the categorical variables by 5. Non-obesity related cancers do not include non-melanoma skin cancer.

Abbreviations: BMI: Body Mass Index; CVD: Cardiovascular disease; HTN: Hypertension; IQR: Interquartile range; MEDEA: “Mortalidad en áreas pequeñas Españolas y Desigualdades Socioeconómicas y Ambientales”; SIDIAP: Information System for Research in Primary Care; T2DM: Type 2 diabetes mellitus.

# Figure S2. Association between body mass index and the risk of obesity-related cancers by ascertainment of incident cardiometabolic conditions, with 95% CIs, minimally- and fully-adjusted models


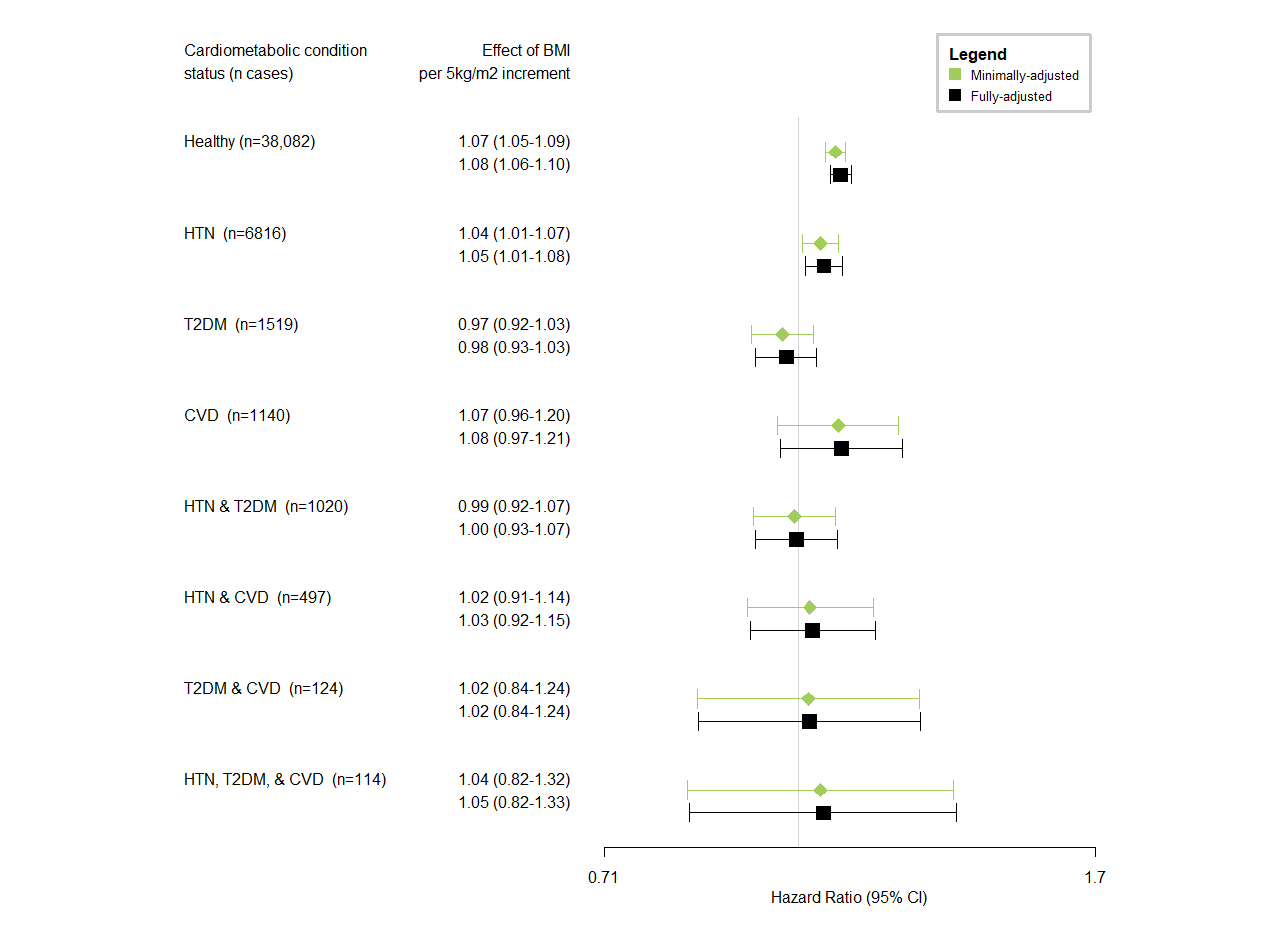


Notes: Both models included BMI as a continuous variable with an interaction term with the time-varying “cardiometabolic conditions” variable. The minimally-adjusted model was only adjusted by sex, and stratified by age (5-year categories). The model fully-adjusted (main) model was adjusted by sex, the geographic region of nationality, the MEDEA deprivation index, smoking status, alcohol intake, and stratified by age (5-year categories).

Abbreviations: BMI: body mass index; CI: confidence interval; CVD: Cardiovascular disease; HTN: Hypertension; KG: kilograms; M: meters; T2DM: Type 2 diabetes mellitus.

# Appendix 2. Description of the supplementary and sensitivity analyses of the primary objective of this study.

In the supplementary analyses of the first objective of this study, we observed that the associations were similar for females and males and for those aged <65 or ≥65 years with one exception (Figures S3 A and B): among those with incident T2DM, a BMI increment of 5 kg/m2 was related to an HR of 0.92 (0.85-0.99) among those aged <65 years, while for those aged ≥65 years it was 1.11 (1.00-1.23). The associations for the site-specific cancers were similar to those of the obesity related-cancers with one specific exception (Figure S4): among those with HTN, the association between BMI and ovary cancer (0.87, 0.74-1.02) was lower than that of BMI and obesity-related cancers (1.05, 1.01-1.08). Our results were robust (the CIs of the sensitivity analyses consistently included the effect estimate of the main model) to six out of seven sensitivity analyses (Figure S5, Table S5, S7).

For the sensitivity analysis in which we considered non-obesity-related cancers as the outcome of interest, we were expecting that the association between BMI and non-obesity-related cancer risk by cardiometabolic conditions would be null or non-positive and that the estimates for cancer risk among sub-groups with the different cardiometabolic conditions would be homogenous relative to the “healthy”. We observed a weak inverse association between BMI (per 5 units increment) and the risk of non-obesity-related cancers (0.92; 0.91-0.94) and associations were similar across most sub-groups, with exception of T2DM and HTN & T2DM, where inverse associations were more pronounced (Table S6). We hypothesize that these consistent inverse associations may be due to the inclusion of respiratory tract and prostate cancers in the non-obesity-related cancer group, which have been inversely associated with BMI in linear models.^1^ Nevertheless, the differential association among individuals with T2DM and those with HTN & T2DM as compared to the “healthy” and other sub-groups could indicate a collider bias.

In the sensitivity analyses of the secondary objective of this study, our results were robust (the CIs of the sensitivity analysis consistently included the effect estimate of the main model) to one sensitivity analysis (Table S8). For the sensitivity analysis in which we considered non-obesity-related cancers as the outcome of interest, the association between overweight/obesity and non-obesity-related cancer risk in absence of cardiometabolic conditions (among the “healthy”) was 0.88 (95%CI: 0.86-0.90) and was lower (and negative) than that of the conditions in absence of overweight/obesity, which were positive. This led to a negative RERI, expectedly contrasting the main analysis, and supports our main findings. However, this was not the case for sub-groups with T2DM and with HTN & T2DM, where the RERIs were similar in direction as in the main analysis (Table S9).

References

1. Recalde M, Davila-Batista V, Díaz Y, et al. Body mass index and waist circumference in relation to the risk of 26 types of cancer: a prospective cohort study of 3.5 million adults in Spain. BMC Medicine. 2021;19(1):10. doi:10.1186/s12916-020-01877-3

# Figure S3. Association between body mass index and the risk of obesity-related cancers by ascertainment of incident cardiometabolic conditions, with 95% CIs, stratified by sex and age

1. Stratification by sex


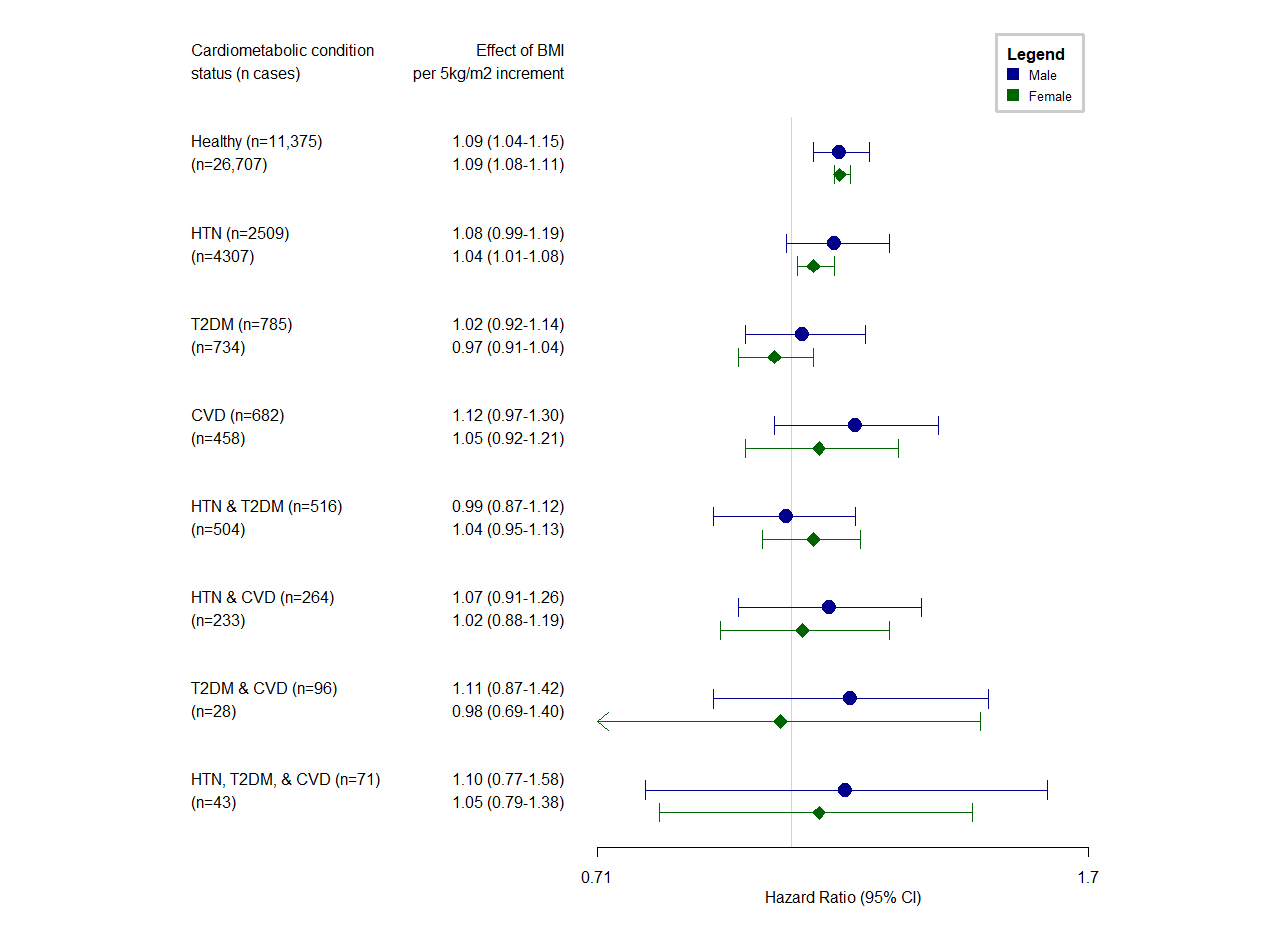


1. Stratification by age


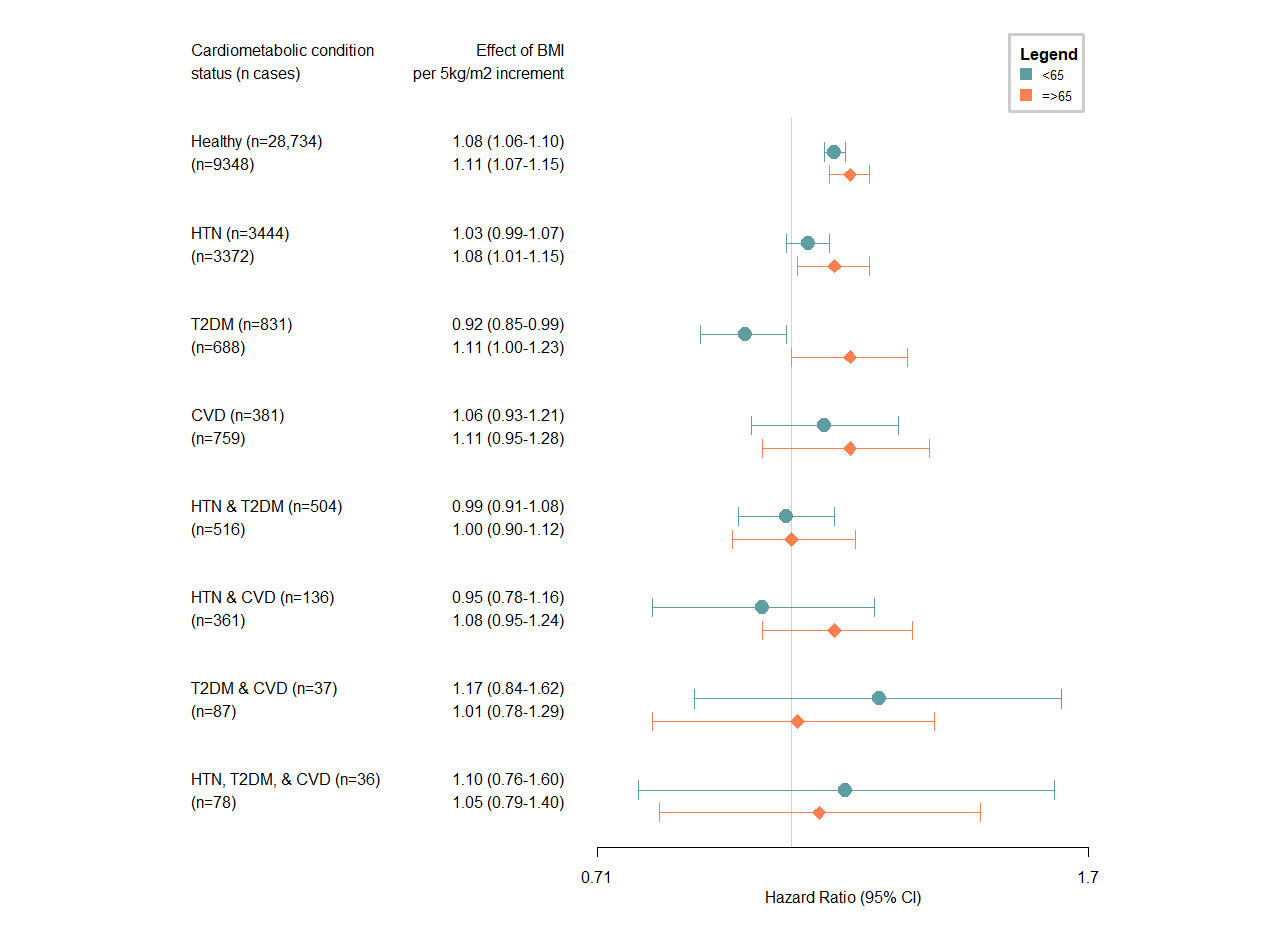


Notes: The models included BMI as a continuous variable with an interaction term with the time-varying “cardiometabolic conditions” variable and were adjusted by sex, the geographic region of nationality, the MEDEA deprivation index, smoking status, alcohol intake, and stratified by age (5-year categories).

Abbreviations: BMI: body mass index; CI: confidence interval; CVD: Cardiovascular disease; HTN: Hypertension; KG: kilograms; M: meters; T2DM: Type 2 diabetes mellitus.

# Figure S4. Association between body mass index and the risk of specific cancer types by ascertainment of incident cardiometabolic conditions, with 95% CI


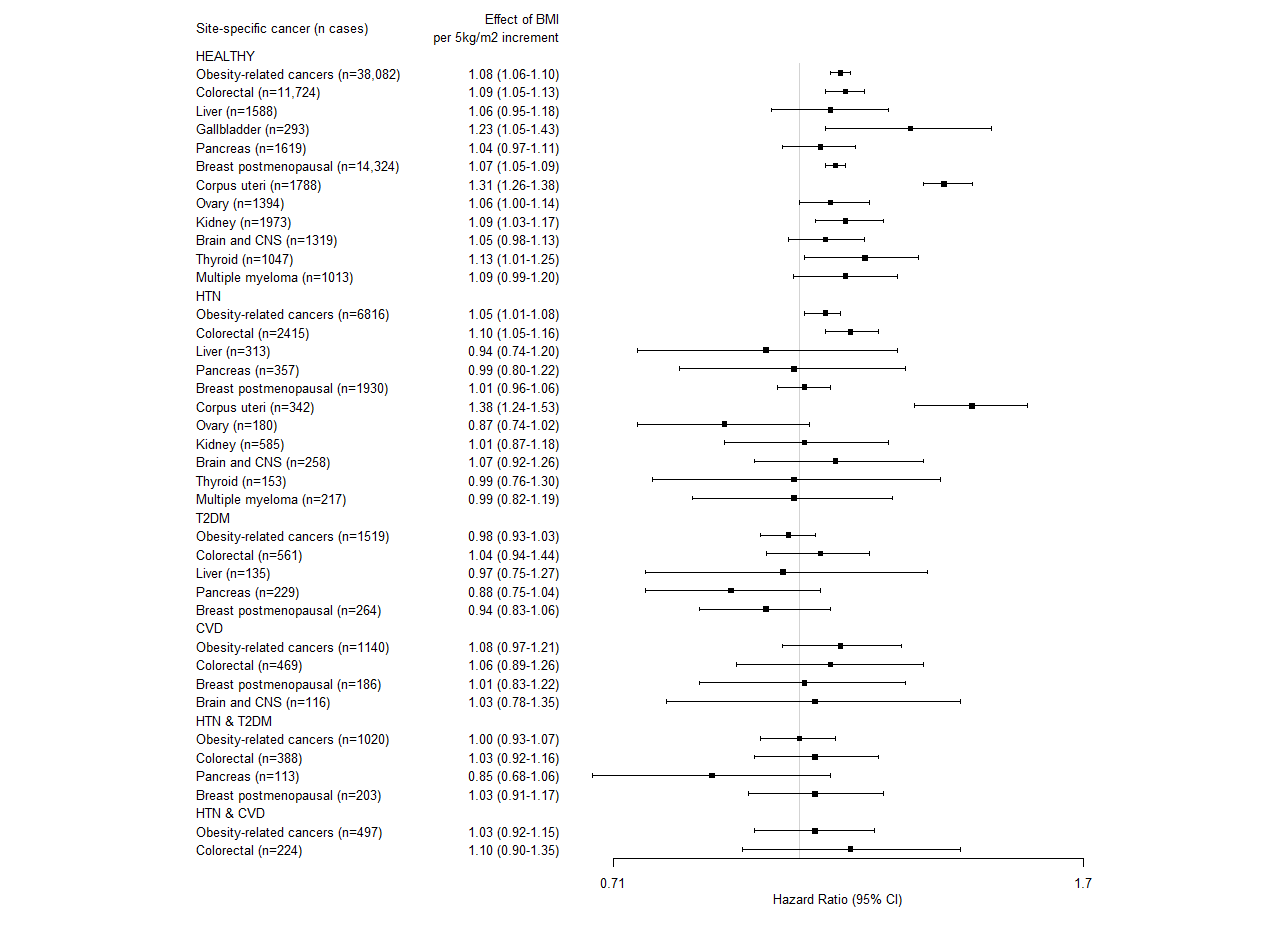


Notes: Separate models were fitted for each site-specific cancer and for the obesity-related cancers outcome. All the models included BMI as a continuous variable with an interaction term with the time-varying “cardiometabolic conditions” variable and were adjusted by sex, the geographic region of nationality, the MEDEA deprivation index, smoking status, alcohol intake, and stratified by age (5-year categories). Brain and CNS include pituitary gland and pineal gland tumors.

Abbreviations: BMI: body mass index; CI: confidence interval; CNS: Central Nervous System; CVD: Cardiovascular disease; HTN: Hypertension; KG: kilograms; M: meters; T2DM: Type 2 diabetes mellitus.

# Figure S5. Sensitivity analyses: association between body mass index and the risk of obesity-related cancers by ascertainment of incident cardiometabolic conditions, with 95% CIs


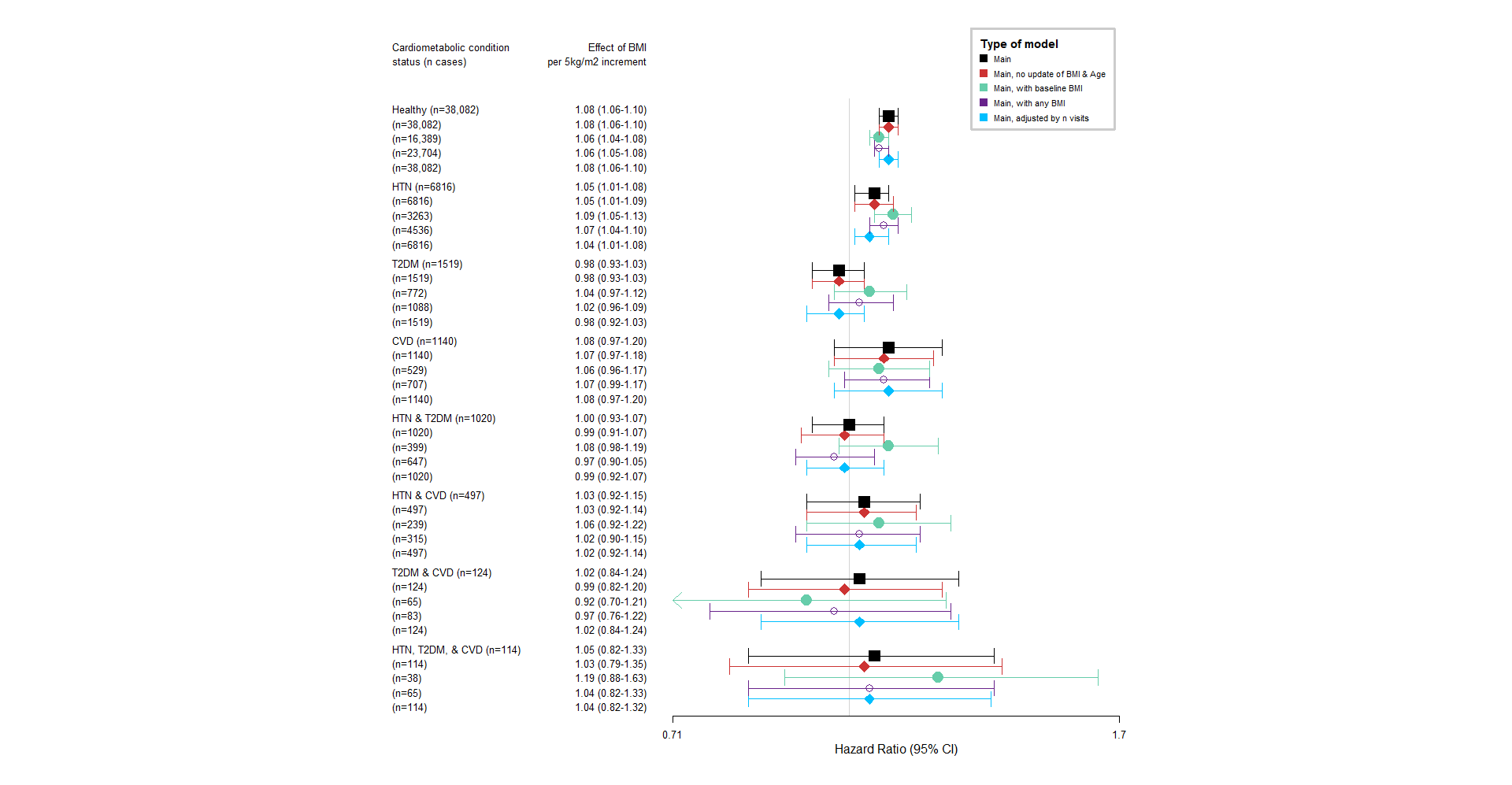


Notes: All the models displayed in this figure included BMI as a continuous variable with an interaction term with the time-varying “cardiometabolic conditions” variable and were adjusted by sex, the geographic region of nationality, the MEDEA deprivation index, smoking status, alcohol intake, and stratified by age (5-year categories). The “Main” model was the one presented in our results as the main analysis. The “Main, no update of BMI & Age” model included the BMI and age of the participants at study entry. The “Main, with baseline BMI” model only included individuals without imputed BMI, who had a real BMI assessment measured before or on the 1st January 2010. The “Main, with any BMI” model included individuals without imputed BMI, but with any BMI assessment (either measured before or on the 1st January 2010 or during follow-up). The “Main, adjusted by n visits” model was also adjusted by the number of visits to primary care centers in the year prior to study entry to account for potentially different health attitudes of the participants.

Abbreviations: BMI: body mass index; CI: confidence interval; CVD: Cardiovascular disease; HTN: Hypertension; KG: kilograms; M: meters; T2DM: Type 2 diabetes mellitus.

# Table S5. Sensitivity analysis: association between body mass index and the risk of obesity-related cancers by ascertainment of incident cardiometabolic conditions, among never smokers, with 95% CIs

|  | HR (95% CI)  Effect of BMI, per 5kg/m^2^ increment | |
| --- | --- | --- |
|  | Main analysis | Sensitivity analysis (among never smokers) |
| Cardiometabolic condition status |  |  |
| Healthy | 1.08 (1.06-1.10) | 1.09 (1.07-1.11) |
| HTN | 1.05 (1.01-1.08) | 1.06 (1.01-1.12) |
| T2DM | 0.98 (0.93-1.03) | 0.99 (0.91-1.08) |
| CVD | 1.08 (0.97-1.21) | 1.10 (0.98-1.22) |
| HTN & T2DM | 1.00 (0.93-1.07) | 1.00 (0.93-1.08) |
| HTN & CVD | 1.03 (0.92-1.15) | 1.04 (0.87-1.23) |
| T2DM & CVD | 1.02 (0.84-1.24) | 0.98 (0.75-1.28) |
| HTN, T2DM, & CVD | 1.05 (0.82-1.33) | 1.05 (0.82-1.34) |

Notes: The models displayed in this table included BMI as a continuous variable with an interaction term with the time-varying “cardiometabolic conditions” variable and were adjusted by sex, the geographic region of nationality, the MEDEA deprivation index, smoking status, alcohol intake, and stratified by age (5-year categories). The “Main analysis” model was the one presented in our results as the main analysis. The “Sensitivity analysis” model only included individuals considered as “never smokers”.

Abbreviations: BMI: body mass index; CI: confidence interval; CVD: Cardiovascular disease; HR: Hazard Ratio; HTN: Hypertension; KG: kilograms; M: meters; T2DM: Type 2 diabetes mellitus

# Table S6. Sensitivity analysis: association between body mass index and the risk of non-obesity-related cancers by ascertainment of incident cardiometabolic conditions, with 95% Cis

|  | HR (95% CI)  Effect of BMI, per 5kg/m^2^ increment | |
| --- | --- | --- |
|  | Main analysis | Sensitivity analysis (non-obesity-related cancers as the outcome) |
| Cardiometabolic condition status |  |  |
| Healthy | 1.08 (1.06-1.10) | 0.92 (0.91-0.94) |
| HTN | 1.05 (1.01-1.08) | 0.90 (0.86-0.94) |
| T2DM | 0.98 (0.93-1.03) | 0.86 (0.81-0.92) |
| CVD | 1.08 (0.97-1.21) | 0.94 (0.87-1.01) |
| HTN & T2DM | 1.00 (0.93-1.07) | 0.83 (0.77-0.90) |
| HTN & CVD | 1.03 (0.92-1.15) | 0.87 (0.79-0.95) |
| T2DM & CVD | 1.02 (0.84-1.24) | 0.86 (0.73-1.00) |
| HTN, T2DM, & CVD | 1.05 (0.82-1.33) | 1.04 (0.89-1.22) |

Notes: The models displayed in this table included BMI as a continuous variable with an interaction term with the time-varying “cardiometabolic conditions” variable and were adjusted by sex, the geographic region of nationality, the MEDEA deprivation index, smoking status, alcohol intake, and stratified by age (5-year categories). The “Main analysis” model was the one presented in our results as the main analysis. The “Sensitivity analysis” model had as an outcome “non-obesity related cancers” instead of “obesity-related cancers”.

Abbreviations: BMI: body mass index; CI: confidence interval; CVD: Cardiovascular disease; HR: Hazard Ratio; HTN: Hypertension; KG: kilograms; M: meters; T2DM: Type 2 diabetes mellitus

# Table S7. Sensitivity analysis: association between body mass index and the risk of obesity-related cancers by ascertainment of incident cardiometabolic conditions, restricting the definition of obesity-related cancers, with 95% CIs

|  | HR (95% CI)  Effect of BMI, per 5kg/m^2^ increment | |
| --- | --- | --- |
|  | Main analysis | Sensitivity analysis (restricting the definition of the outcome) |
| Cardiometabolic condition status |  |  |
| Healthy | 1.08 (1.06-1.10) | 1.08 (1.06-1.10) |
| HTN | 1.05 (1.01-1.08) | 1.04 (1.01-1.08) |
| T2DM | 0.98 (0.93-1.03) | 0.98 (0.92-1.03) |
| CVD | 1.08 (0.97-1.21) | 1.08 (0.98-1.20) |
| HTN & T2DM | 1.00 (0.93-1.07) | 0.99 (0.92-1.07) |
| HTN & CVD | 1.03 (0.92-1.15) | 1.04 (0.93-1.16) |
| T2DM & CVD | 1.02 (0.84-1.24) | 1.04 (0.85-1.27) |
| HTN, T2DM, & CVD | 1.05 (0.82-1.33) | 1.04 (0.82-1.31) |

Notes: The models displayed in this table included BMI as a continuous variable with an interaction term with the time-varying “cardiometabolic conditions” variable and were adjusted by sex, the geographic region of nationality, the MEDEA deprivation index, smoking status, alcohol intake, and stratified by age (5-year categories). The “Main analysis” model was the one presented in our results as the main analysis. The “Sensitivity analysis” model had a more restrictive definition of the outcome (for corpus uteri we considered only C54 and C54.1 as codes of interest, and we excluded brain and CNS cancer from the obesity-related cancer definition, as only meningioma is considered an obesity-related cancer in this broad group).

Abbreviations: BMI: body mass index; CI: confidence interval; CVD: Cardiovascular disease; HR: Hazard Ratio; HTN: Hypertension; KG: kilograms; M: meters; T2DM: Type 2 diabetes mellitus.

# Figure S6. Association between incident cardiometabolic conditions and the risk of obesity-related cancers, with 95% CIs

#


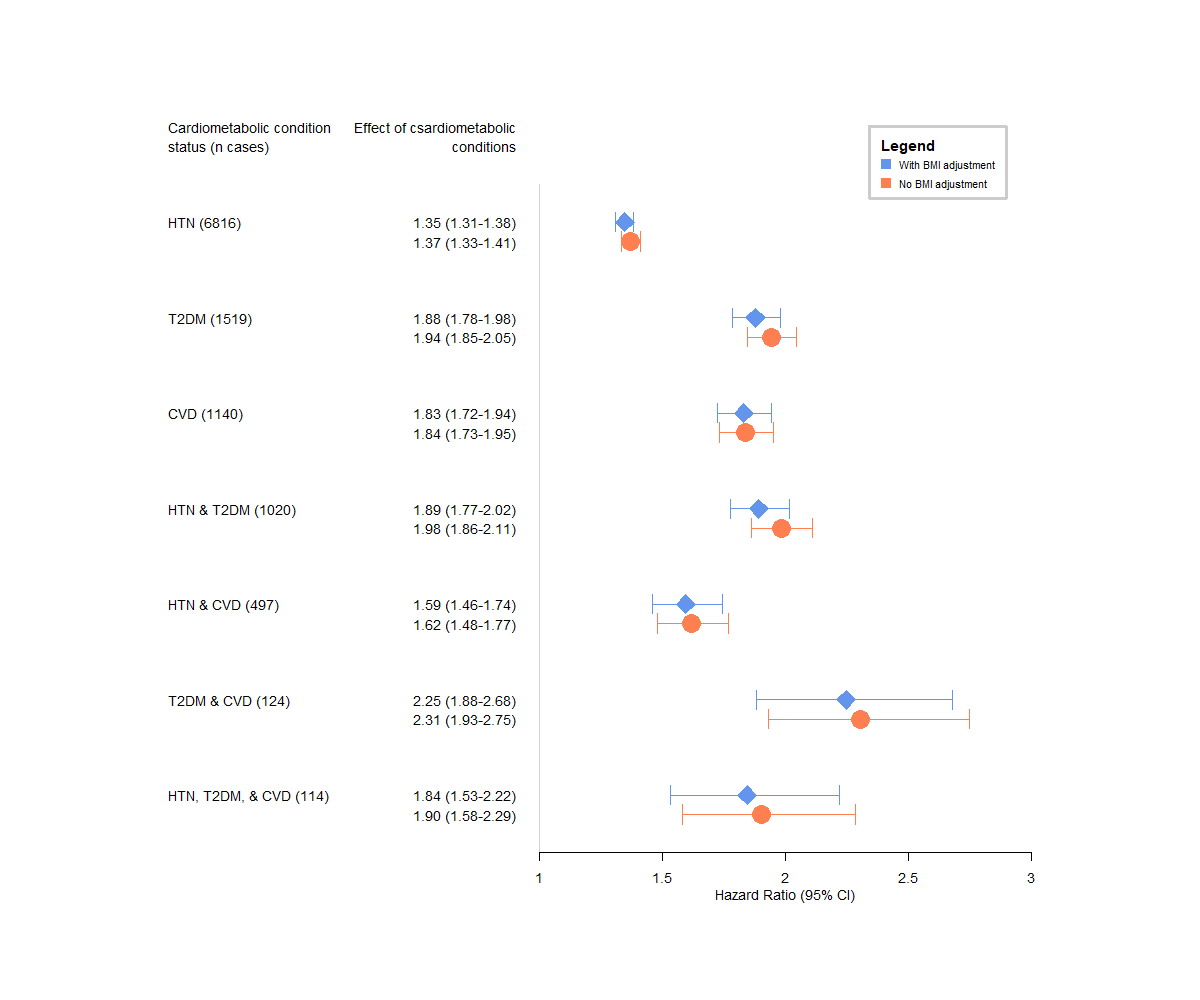


Notes: Both models included the time-varying “cardiometabolic conditions” variable and were adjusted by sex, the geographic region of nationality, the MEDEA deprivation index, smoking status, alcohol intake, and stratified by age (5-year categories). “With BMI adjustment” was also adjusted by BMI.

Abbreviations: BMI: Body mass index; CI: confidence interval; CVD: Cardiovascular disease; HTN: Hypertension; T2DM: Type 2 diabetes mellitus.

# Table S8. Sensitivity analysis: Relative risk of obesity-related cancers due to interaction between overweight/obesity (BMI≥25 kg/m2) and incident cardiometabolic conditions, among never smokers, with 95% CIs

|  | **HTN**  **HR (95% CI)** | | **T2DM**  **HR (95% CI)** | | **CVD**  **HR (95% CI)** | | **HTN & T2DM**  **HR (95% CI)** | | **HTN & CVD**  **HR (95% CI)** | | **T2DM & CVD**  **HR (95% CI)** | | **HTN, T2DM, & CVD**  **HR (95% CI)** | |
| --- | --- | --- | --- | --- | --- | --- | --- | --- | --- | --- | --- | --- | --- | --- |
|  | **Main** | **Sensitivity** | **Main** | **Sensitivity** | **Main** | **Sensitivity** | **Main** | **Sensitivity** | **Main** | **Sensitivity** | **Main** | **Sensitivity** | **Main** | **Sensitivity** |
| **BMI<25 kg/m^2^, *“healthy”*** | 1 (ref) | 1 (ref) | 1 (ref) | 1 (ref) | 1 (ref) | 1 (ref) | 1 (ref) | 1 (ref) | 1 (ref) | 1 (ref) | 1 (ref) | 1 (ref) | 1 (ref) | 1 (ref) |
| **BMI ≥25 kg/m^2^, *“healthy”*** | 1.11  (1.06-1.16) | 1.12  (1.08-1.17) | 1.11  (1.06-1.16) | 1.12  (1.08-1.17) | 1.11  (1.06-1.16) | 1.12  (1.08-1.17) | 1.11  (1.06-1.16) | 1.12  (1.08-1.17) | 1.11  (1.06-1.16) | 1.12  (1.08-1.17) | 1.11  (1.06-1.16) | 1.12  (1.08-1.17) | 1.11  (1.06-1.16) | 1.12  (1.08-1.17) |
| **BMI<25 kg/m^2^, with condition** | 1.40  (1.29-1.53) | 1.38  (1.22-1.57) | 2.18  (1.91-2.50) | 2.19  (1.85-2.60) | 1.76  (1.44-2.16) | 1.75  (1.41-2.17) | 2.25  (1.82-2.77) | 2.26  (1.79-2.86) | 1.63  (1.35-1.96) | 1.64  (1.26-2.14) | 2.09  (1.18-3.71) | 1.82  (0.96-3.43) | 1.76  (0.87-3.54) | 2.00  (0.99-4.02) |
| **BMI ≥25 kg/m^2^, with condition (joint effect)** | 1.49  (1.44-1.55) | 1.53 (1.46-1.59) | 2.07  (1.93-2.22) | 2.07  (1.89-2.27) | 2.07  (1.84-2.32) | 2.06  (1.81-2.35) | 2.12  (1.97-2.27) | 2.15  (1.96-2.35) | 1.78  (1.60-1.98) | 1.79  (1.56-2.06) | 2.57  (2.08-3.17) | 2.39  (1.77-3.21) | 2.10  (1.70-2.59) | 2.25  (1.73-2.93) |
| **RERI** | -0.02  (-0.06, 0.02) | 0.02  (-0.02, 0.06) | -0.22  (-0.29, -0.16) | -0.25  (-0.29, -0.21) | 0.19  (0.09, 0.30) | 0.19  (0.15, 0.23) | -0.24  (-0.28, -0.20) | -0.24  (-0.28, -0.20) | 0.04  (-0.07, 0.15) | 0.03  (-0.01, 0.06) | 0.36  (0.32, 0.40) | 0.45  (0.41, 0.48) | 0.23  (0.02, 0.44) | -0.12  (-0.16, -0.09) |

Notes: The models were adjusted by sex, the geographic region of nationality, the MEDEA deprivation index, smoking status, alcohol intake, and stratified by age (5-year categories). A RERI of 0 denotes lack of additive interaction. The “Main” model was the one presented in our results as the main analysis. The “Sensitivity” model only included individuals considered as “never smokers”.

Abbreviations: BMI: Body mass index; CI: confidence interval; CVD: Cardiovascular disease; HR: hazard ratio; HTN: Hypertension; RERI: relative excess risk due to interaction; T2DM: Type 2 diabetes mellitus.

# Table S9. Sensitivity analysis: Relative risk of non-obesity-related cancers due to interaction between overweight/obesity (BMI≥25 kg/m2) and incident cardiometabolic conditions, with 95% CIs

|  | **HTN**  **HR (95% CI)** | | **T2DM**  **HR (95% CI)** | | **CVD**  **HR (95% CI)** | | **HTN & T2DM**  **HR (95% CI)** | | **HTN & CVD**  **HR (95% CI)** | | **T2DM & CVD**  **HR (95% CI)** | | **HTN, T2DM, & CVD**  **HR (95% CI)** | |
| --- | --- | --- | --- | --- | --- | --- | --- | --- | --- | --- | --- | --- | --- | --- |
|  | **Main** | **Sensitivity** | **Main** | **Sensitivity** | **Main** | **Sensitivity** | **Main** | **Sensitivity** | **Main** | **Sensitivity** | **Main** | **Sensitivity** | **Main** | **Sensitivity** |
| **BMI<25 kg/m^2^, *“healthy”*** | 1 (ref) | 1 (ref) | 1 (ref) | 1 (ref) | 1 (ref) | 1 (ref) | 1 (ref) | 1 (ref) | 1 (ref) | 1 (ref) | 1 (ref) | 1 (ref) | 1 (ref) | 1 (ref) |
| **BMI ≥25 kg/m^2^, *“healthy”*** | 1.11  (1.06-1.16) | 0.88  (0.86-0.90) | 1.11  (1.06-1.16) | 0.88  (0.86-0.90) | 1.11  (1.06-1.16) | 0.88  (0.86-0.90) | 1.11  (1.06-1.16) | 0.88  (0.86-0.90) | 1.11  (1.06-1.16) | 0.88  (0.86-0.90) | 1.11  (1.06-1.16) | 0.88  (0.86-0.90) | 1.11  (1.06-1.16) | 0.88  (0.86-0.90) |
| **BMI<25 kg/m^2^, with condition** | 1.40  (1.29-1.53) | 1.34  (1.25-1.43) | 2.18  (1.91-2.50) | 1.72  (1.53-1.93) | 1.76  (1.44-2.16) | 1.96  (1.80-2.15) | 2.25  (1.82-2.77) | 1.81  (1.56-2.11) | 1.63  (1.35-1.96) | 1.77  (1.47-2.12) | 2.09  (1.18-3.71) | 2.32  (1.66-3.23) | 1.76  (0.87-3.54) | 1.55  (1.04-2.31) |
| **BMI ≥25 kg/m^2^, with condition (joint effect)** | 1.49  (1.44-1.55) | 1.10  (1.06-1.14) | 2.07  (1.93-2.22) | 1.25  (1.18-1.33) | 2.07  (1.84-2.32) | 1.62  (1.53-1.71) | 2.12  (1.97-2.27) | 1.21  (1.13-1.29) | 1.78  (1.60-1.98) | 1.47  (1.34-1.61) | 2.57  (2.08-3.17) | 1.86  (1.59-2.18) | 2.10  (1.70-2.59) | 1.46  (1.25-1.72) |
| **RERI** | -0.02  (-0.06, 0.02) | -0.12  (-0.14, -0.09) | -0.22  (-0.29, -0.16) | -0.35  (-0.37, -0.32) | 0.19  (0.09, 0.30) | -0.22  (-0.25, -0.20) | -0.24  (-0.28, -0.20) | -0.48  (-0.51, -0.46) | 0.04  (-0.07, 0.15) | -0.17  (-0.20, -0.15) | 0.36  (0.32, 0.40) | -0.33  (-0.36, -0.31) | 0.23  (0.02, 0.44) | 0.12  (0.10, 0.15) |

Notes: The models were adjusted by sex, the geographic region of nationality, the MEDEA deprivation index, smoking status, alcohol intake, and stratified by age (5-year categories). A RERI of 0 denotes lack of additive interaction. The “Main” model was the one presented in our results as the main analysis. The “Sensitivity3” model had as an outcome “non-obesity related cancers” instead of “obesity-related cancers”.

Abbreviations: BMI: Body mass index; CI: confidence interval; CVD: Cardiovascular disease; HR: hazard ratio; HTN: Hypertension; RERI: relative excess risk due to interaction; T2DM: Type 2 diabetes mellitus.

# Appendix 3. STROBE Statement checklist

|  | **Item No** | **Recommendation** | **Page No** |
| --- | --- | --- | --- |
| **Title and abstract** | 1 | (*a*) Indicate the study’s design with a commonly used term in the title or the abstract | 1 |
|  |  | (*b*) Provide in the abstract an informative and balanced summary of what was done and what was found | 3 |
| **Introduction** | | |  |
| Background/rationale | 2 | Explain the scientific background and rationale for the investigation being reported | 4 |
| Objectives | 3 | State specific objectives, including any prespecified hypotheses | 4 |
| **Methods** | | |  |
| Study design | 4 | Present key elements of study design early in the paper | 5 |
| Setting | 5 | Describe the setting, locations, and relevant dates, including periods of recruitment, exposure, follow-up, and data collection | 5 |
| Participants | 6 | (*a*) Give the eligibility criteria, and the sources and methods of selection of participants. Describe methods of follow-up | 5 |
|  |  | (*b*) For matched studies, give matching criteria and number of exposed and unexposed | - |
| Variables | 7 | Clearly define all outcomes, exposures, predictors, potential confounders, and effect modifiers. Give diagnostic criteria, if applicable | 5-7 |
| Data sources/ measurement | 8* | For each variable of interest, give sources of data and details of methods of assessment (measurement). Describe comparability of assessment methods if there is more than one group | 5 |
| Bias | 9 | Describe any efforts to address potential sources of bias | 8-9 |
| Study size | 10 | Explain how the study size was arrived at | - |
| Quantitative variables | 11 | Explain how quantitative variables were handled in the analyses. If applicable, describe which groupings were chosen and why | 7-9 |
| Statistical methods | 12 | (*a*) Describe all statistical methods, including those used to control for confounding | 7-9 |
|  |  | (*b*) Describe any methods used to examine subgroups and interactions | 7-9 |
|  |  | (*c*) Explain how missing data were addressed | 6, 7-9 |
|  |  | (*d*) If applicable, explain how loss to follow-up was addressed | 8 |
|  |  | (*e*) Describe any sensitivity analyses | 7-9 |
| **Results** | | |  |
| Participants | 13* | (a) Report numbers of individuals at each stage of study—eg numbers potentially eligible, examined for eligibility, confirmed eligible, included in the study, completing follow-up, and analysed | 9 |
|  |  | (b) Give reasons for non-participation at each stage | 9 |
|  |  | (c) Consider use of a flow diagram | Fig 1 |
| Descriptive data | 14* | (a) Give characteristics of study participants (eg demographic, clinical, social) and information on exposures and potential confounders | 9 |
|  |  | (b) Indicate number of participants with missing data for each variable of interest | Table 1. |
|  |  | (c) Summarise follow-up time (eg, average and total amount) | 10 Table 1. |
| Outcome data | 15* | Report numbers of outcome events or summary measures over time | 10 Table 1. |
| Main results | 16 | (*a*) Give unadjusted estimates and, if applicable, confounder-adjusted estimates and their precision (eg, 95% confidence interval). Make clear which confounders were adjusted for and why they were included | 10 Supp. |
|  |  | (*b*) Report category boundaries when continuous variables were categorized | 9 Table 1. |
|  |  | (*c*) If relevant, consider translating estimates of relative risk into absolute risk for a meaningful time period | 10-11 Table 2B |
| Other analyses | 17 | Report other analyses done—eg analyses of subgroups and interactions, and sensitivity analyses | Supp. |
| **Discussion** | | |  |
| Key results | 18 | Summarise key results with reference to study objectives | 11 |
| Limitations | 19 | Discuss limitations of the study, taking into account sources of potential bias or imprecision. Discuss both direction and magnitude of any potential bias | 12-13 |
| Interpretation | 20 | Give a cautious overall interpretation of results considering objectives, limitations, multiplicity of analyses, results from similar studies, and other relevant evidence | 11-13 |
| Generalisability | 21 | Discuss the generalisability (external validity) of the study results | 12 |
| **Other information** | | |  |
| Funding | 22 | Give the source of funding and the role of the funders for the present study and, if applicable, for the original study on which the present article is based | 19 |
